# Ferrocenoylation of Uracil Derivatives: Study of Reaction Regioselectivity and Biological Activity

**DOI:** 10.3390/molecules31061054

**Published:** 2026-03-23

**Authors:** Jasmina Lapić, Ivana Kuzman, Ruža Frkanec, Leo Frkanec, Senka Djaković

**Affiliations:** 1 Faculty of Food Technology and Biotechnology, University of Zagreb, Pierottijeva 6, 10000 Zagreb, Croatia; jasmina.lapic@pbf.unizg.hr (J.L.);; 2 Centre for Research and Knowledge Transfer in Biotechnology, University of Zagreb, Rockefellerova 10, 10000 Zagreb, Croatia; ruza.frkanec@unizg.hr; 3 Rudjer Bošković Institute, Bijenička 54, 10000 Zagreb, Croatia; frkanec@irb.hr

**Keywords:** uracil derivatives, ferrocenoylation, biological activity, regioselectivity, model reaction

## Abstract

The *N*-ferrocenoylation of uracil was studied to evaluate regioselectivity and optimise preparation protocols. Regioselectivity was monitored under various reaction conditions, with particular attention paid to the effects of the solvent and the base. Reactions in DMF were regiospecific, yielding only the *N*1 product, while reactions in CH_3_CN produced both *N*1 and *N*1/*N*3 products, with ratios depending on the reaction conditions. The highest yield of *N*1/*N*3-diferrocenoyl uracil was achieved with an extended reaction time of 90 min using uracil and triethylamine. Optimised conditions were applied to *C*5-uracil derivatives, producing *N*1 and *N*1/*N*3 products. Regioselectivity and *N*-substitution were confirmed by NMR, and solvent effects were supported by quantum chemical calculations. The resulting ferrocene–pyrimidine conjugates exhibited oxidative and immunomodulatory activity, highlighting their biological potential.

## 1. Introduction

Medicinal chemistry, as a tool for developing and synthesising new compounds with potentials as drugs, has, for some time, focused on compounds that are hybrids of an organometallic fragment and another bioactive molecule. Organometallic fragments can introduce a wide range of new pharmacophores with unique interactions at target sites, leading to the discovery of drugs with novel mechanisms of action. One of the most interesting members of the metallocene family is ferrocene, which contains Fe(II) and two cyclopentadienyl ligands in a rigid sandwich structure. It is stable in physiological media and possesses lipophilic properties that allow it to cross cell membranes efficiently [1,2,3,4]. It can also be relatively easily derivatised, enabling its incorporation into various carriers [5,6,7,8,9]. Synthetic strategies for introducing ferrocene into pharmacophores involve either replacing the phenyl (ferrocyphene), alkyl (ferroquine), or heterocyclic ring of a known drug with a ferrocenyl group or attaching this group to various bioactive compounds, natural products, peptides, steroids, betulins, etc., via a functional group. Electronic delocalisation through the conjugation of ferrocenyl with bioactive molecules is favourable for oxidation, and our research group has linked the two fragments, ferrocene and nucleobase, via a carbonyl group. As both FcH derivatives and [Fc]^+^ have long been known for their anticancer and antimicrobial activities, one of the key parameters is the redox stability of both redox forms in biological media. Ferrocene chemistry involves the simple derivatisation of one or both cyclopentadienyl rings, which have stable redox forms (Fe(II) ↔ Fe(III)), and these forms can be easily interconverted under biological conditions without destruction or loss of function. The flexible structure and favourable physical properties of ferrocene drugs provide a solid foundation for developing sophisticated therapeutic strategies for precise, localised chemotherapy [10,11,12].

Nucleosides and their analogues play an important role in drug discovery and development due to their notable chemotherapeutic activities. In these drug molecules, the sugar moiety is often modified (e.g., in AZT or gemcitabine) or completely replaced (e.g., in abacavir and acyclovir) [13,14,15,16,17]. Studies on various ferrocene derivatives suggest that cancer cell cytotoxicity results from oxidation to the ferrocenium ion, which facilitates the formation of reactive oxygen species (ROS) that damage genetic material and induce apoptosis. In developing new biomolecules based on conjugates of ferrocene and nucleobases, the focus is on mimicking the parent structure of nucleosides and preserving the potential biological activity of the prepared compounds [18,19,20,21]. For the synthesis of nucleoside derivatives, the *N*-alkylation of nucleobases is often performed with different carbon electrophiles. However, the reactivity of nucleobases is often limited by their lower nucleophilicity and increased acidity of NH groups [13,22]. Replacing the sugar fragment of nucleosides with a redox-active, metal-containing ferrocenyl group could provide access to electron-conducting, self-assembling polymers, with applications in biology, medicine, and nanotechnology. The binding of substituents to the ferrocene molecule is enabled by the rotation of its cyclopentadienyl rings, and ferrocene acquires biological properties when a biological molecule such as a nucleobase is attached. This biological activity is an increasingly common research topic, with the first step being the synthesis of bioconjugates. Ferrocene is attached to the nitrogen base as an *N*-substituent, and the binding site determines the biological properties of the compound itself [23,24,25,26,27,28].

**Figure 1:**
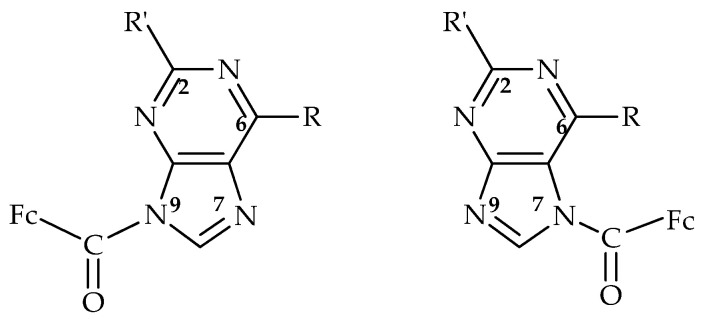
Structure of *N*7- and *N*9-ferrocenoyl purine derivatives.

In our previous research on the preparation of organometallic ferrocene derivatives, we tested the regioselectivity of these reactions, which included both pyrimidine [29] and purine bases [30]. The bioconjugates were prepared by reacting ferrocenoyl chloride with sodium hydride in dimethylformamide. This reaction is regiospecific for pyrimidine derivatives, resulting in the formation of only *N*1 isomers [29], whereas purine derivatives exhibit different regioselectivity, with both *N*7 and *N*9 isomers formed. By introducing a suitable substituent at the *C*6 position of the purine ring, the ratio of the isomeric products *N*7 and *N*9 can be adjusted, i.e., the regioselectivity of the ferrocenoylation reaction can be modulated (Figure 1) [30].

**Figure 2:**
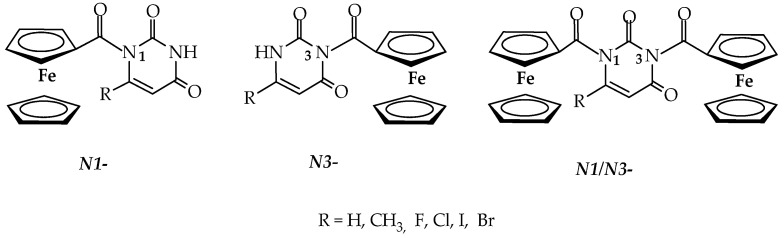
Structure of *N*1-, *N*3- and *N*1/*N*3-ferrocenoyl pyrimidine derivatives.

This paper presents further research into the regioselectivity of the *N*-ferrocenoylation reaction of pyrimidine bases by varying reaction conditions. A model reaction on uracil was carried out to optimise the reaction conditions, focusing on the solvent and base, and their effects on regioselectivity were monitored. The optimised conditions were then applied to uracil derivatives bearing either electron-donating or electron-accepting groups at the *C*5 and *C*6 positions (Figure 2). The regioselectivity of the reactions was assessed using the ^1^H NMR technique. Density functional theory (DFT) calculations were performed to elucidate the mechanistic details underlying the observed regioselectivity. Pyrimidine-containing compounds are attractive candidates for drug development due to their diverse properties and unique structures, which can be readily modified [31]. This structural flexibility enables the design of a wide range of pyrimidine-based drugs with various biological activities and functions. The synthesised compounds were analysed for their oxidative and immunostimulatory activity.

## 2. Results and Discussion

### 2.1. Synthesis: Model Reaction, Regioselectivity, and DFT

Pyrimidine nucleobases and their derivatives substituted at the *C*5 position of the pyrimidine ring are characterised by potent biological activity and have been investigated as antiviral agents, nonradioactive fluorescent markers for DNA, and antitumor agents [26,32]. In addition to their biological activity, the hybrids also exhibit interesting electrochemical properties [33]. To mimic the natural structure of nucleosides and preserve the potential biological activity of the synthesised compounds, the ferrocene moiety must be selectively substituted at the *N*1 position of the base in place of the sugar moiety. Our research group has developed a one-step synthetic route for the preparation of the ferrocenoyl derivatives of pyrimidine and purine nucleobases containing a carbonyl group as a chemical spacer [29,30]. In previous studies, the reaction of pyrimidine bases (uracil, 5-fluorouracil, and thymine) with NaH and ferrocenoyl chloride in *N*,*N*-dimethylformamide (DMF) was investigated, and only an *N*1 product was observed [29]. In a similar reaction between a *C*6-substituted purine ring (R = NH_2_, Me, NHBz, and OBz) and ferrocenoyl chloride, two products were isolated, *N*7- and *N*9-ferrocenoylated compounds. We found that the ferrocenoylation of pyrimidine nucleobase derivatives is regiospecific, forming only *N*1 copolymers, whereas for purine derivatives, the reaction is regioselective, forming two products, *N*7 and *N*9, with the ratio in the reaction mixture depending on the *C*6 substituent on the purine ring [30]. In light of these results, a model reaction was performed on uracil to test the regioselectivity of the ferrocene–pyrimidine coupling reaction. In this work, the reaction progress was monitored under various conditions, including different acylating agents [ferrocenoyl chloride (**1**), ferrocenoylethyl carbonate (**2**), and ferrocenoyl benzotriazole (**3**)], solvents (*N*,*N*-dimethylformamide, acetonitrile, and 1,4-dioxane), bases (sodium hydride, potassium carbonate, and triethylamine), and reaction times.

**Scheme 1:**
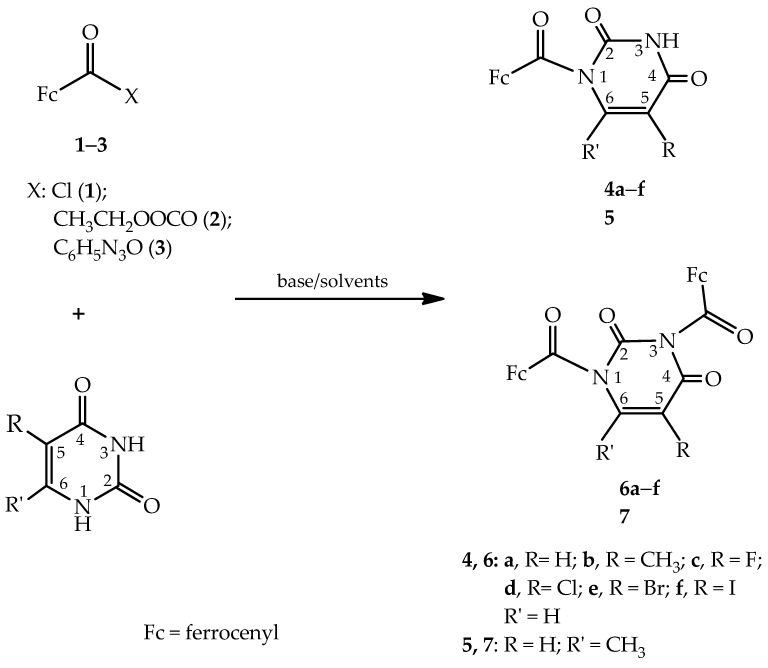
Ferrocenoylation of uracil derivatives; base: NaH or K_2_CO_3_ or Et_3_N; solvents: DMF or CH_3_CN or 1,4-dioxane.

The preparation of ferrocene nucleobase derivatives is based on coupling the organometallic moiety and the heterocyclic pyrimidine base via the carbonyl group as a bridge (Scheme 1). The starting compounds, ferrocenoyl chloride (**1**), ferrocenoylethyl carbonate (**2**), and ferrocenoyl benzotriazole (**3**), were synthesised according to previously described procedures [29,34]. Reaction of the nucleobase with NaH, K_2_CO_3_, or Et_3_N in DMF, CH_3_CN, or 1,4-dioxane leads to formation of a pyrimidine base salt. This is followed by the reaction of the pyrimidine base salt with the acylating agents, which proceeds rapidly and changes colour depending on the acylating agent used. The nucleobase salt is a strong nucleophile in reaction with acylating agents due to the negative charge on the nitrogen atom, which favours an S_N_2-like mechanism.

When ferrocenyl chloride (**1**) is used as an acylating agent with NaH as the base, and the reactions are carried out in DMF, only the *N*1 product is formed (Table 1, line 1). Replacing NaH with K_2_CO_3_ or Et_3_N in the same solvent also yields the *N*1 product only (Table 1, lines 2 and 3). Extending the reaction time to 60 or 90 min, regardless of the base used, results in the formation of the *N*1 product only, but with increased yield (Table 1, lines 4–9). The *N*-ferrocenylation reactions of uracil carried out in CH_3_CN result in a change in the reaction profile, with the formation of *N*1/*N*3-product **6a** in addition to the *N*1 product **4a** (Table 1, lines 10–18). The regioselectivity depends strongly on the type of base and the duration of the uracil salt-forming reaction. NaH and K_2_CO_3_ continue to favour the formation of product **4a**, whereas Et_3_N reduces the selectivity and increases the proportion of **6a** (Table 1, lines 10–18). Extending the reaction time of uracil with the base in CH_3_CN further promotes the formation of bis-products, with the highest yield obtained using Et_3_N after 90 min of reaction (Table 1, line 18). In 1,4-dioxane, the reaction is significantly less efficient, and the products (**4a** and **6a**) were obtained only in the presence of Et_3_N and in low yields (Table 1, lines 21 and 22). In reactions with the other two acylating agents, ferrocenoyl ethyl carbonate (**2**) and ferrocenoyl benzotriazole (**3**) (Tables S1 and S2), the yields of *N*1 products (**4a**) are very low, and *N*1/*N*3 (**6a**) is not formed under any reaction conditions. Previous studies indicate the formation of the *N*1 isomer with a slight excess of the acylating agent, while increasing the excess leads to the formation of the *N*1/*N*3 product. Therefore, the course and regioselectivity of the reaction with a higher proportion of the acylating agent (ferrocenoyl chloride, **1**) at a 1:2.4 ratio were investigated. In DMF, only one product, the *N*1 regioisomer, was formed, while in the reaction carried out in CH_3_CN, the yields of both products increased, but their ratio changed only slightly. The *N*-ferrocenoylation reaction was also tested using a twofold molar excess of the base relative to uracil. The results showed that neither the reaction yield nor the ratio of mono- to bis-ferrocenoylated products changed significantly.

The reaction with *N*1-protected uracil (*N*1 product, **4a**) was also performed, yielding a significant amount of *N*1/*N*3 product (75%) using Et_3_N in CH_3_CN. These results indicate that the precise choice of solvent, base, and reaction time for uracil salt formation is crucial to control the regioselectivity and yield of the *N*-ferrocenoylation reaction of uracil.

**Figure 3:**
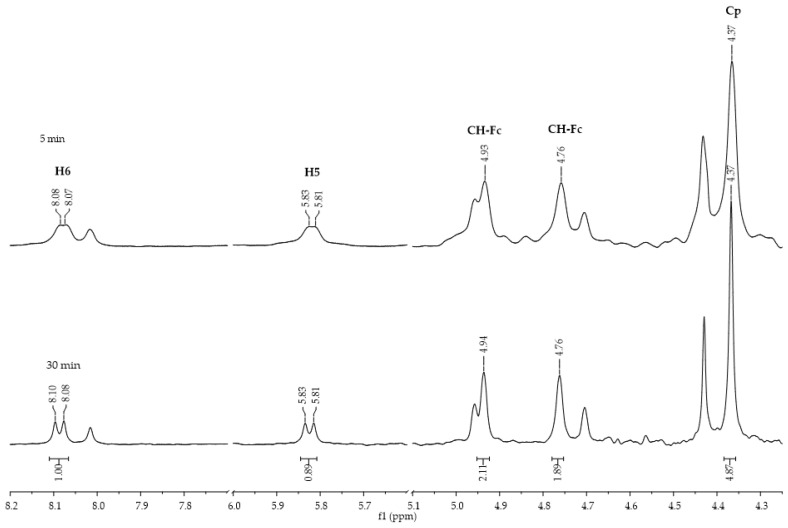
^1^H{^13^C} NMR spectra of the reaction of ferrocenoyl chloride (**1**) and uracil in DMF-*d*_7_.

The reactions were also monitored in situ by ^1^H NMR spectroscopy to determine the proportion of each product. The experiment involved continuous monitoring of the reaction from the first minute to completion, with the fraction of regioisomers formed determined from the integrals of the peaks assigned to each product. First, the progress of the reaction between ferrocenoyl chloride (**1**) and uracil, using NaH, was monitored in DMF-*d*_7_. During this reaction, only the formation of the *N*1 product (**4a**) was observed (Figure 3).

**Figure 4:**
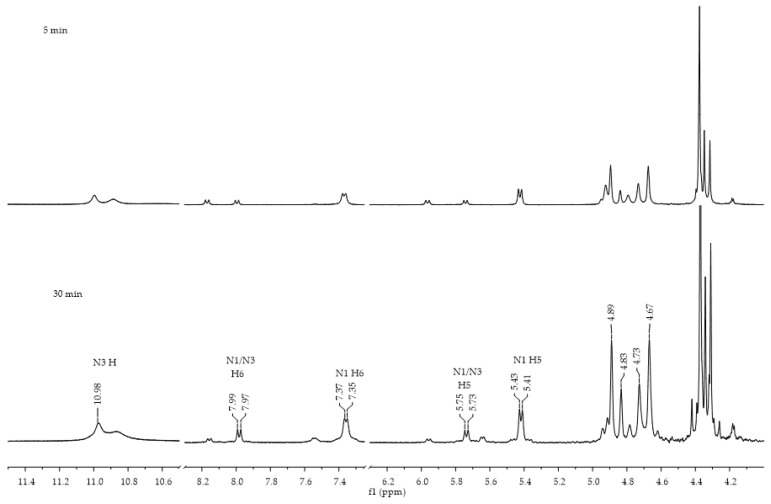
^1^H{^13^C} NMR spectra of the reaction of ferrocenoyl chloride (**1**) and uracil in CH_3_CN-*d*_3_ with Et_3_N as base.

The reaction between ferrocenoyl chloride (**1**) and Et_3_N as the base in CH_3_CN as the solvent was also monitored in situ by ^1^H NMR spectroscopy. At the start of the reaction, the chemical shifts in both products, *N*1 (**4a**) and *N*1/*N*3 (**6a**), are visible on the spectrum, and their proportions do not change significantly during the remainder of the reaction (Figure 4). Chemical shifts characteristic of uracil compounds are also present in the spectrum. These NMR studies indicate that the reactions are very fast and occur instantaneously. This experiment confirmed the synthetic results and the proportion of individual isomers in the reaction mixture.

**Figure 5:**
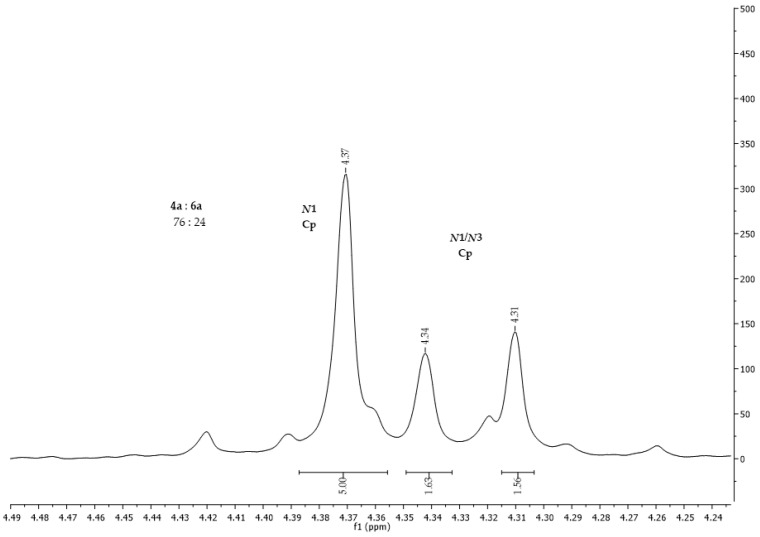
The fraction of *N*1 and *N*1/*N*3 products formed in the reaction of ferrocenoyl chloride (**1**) and uracil in CH_3_CN with Et_3_N as base (90 min of reaction).

The percentage of products formed can be calculated from the ^1^H NMR spectrum (Figure 5). In the reaction between ferrocenoyl chloride (**1**) and uracil, carried out in CH_3_CN with Et_3_N as the base and with a prolonged reaction time of 90 min, 76% of *N*1-ferrocenoyl uracil (**4a**) and 24% of *N*1/*N*3-diferrocenoyl uracil (**6a**) were obtained.

**Scheme 2:**
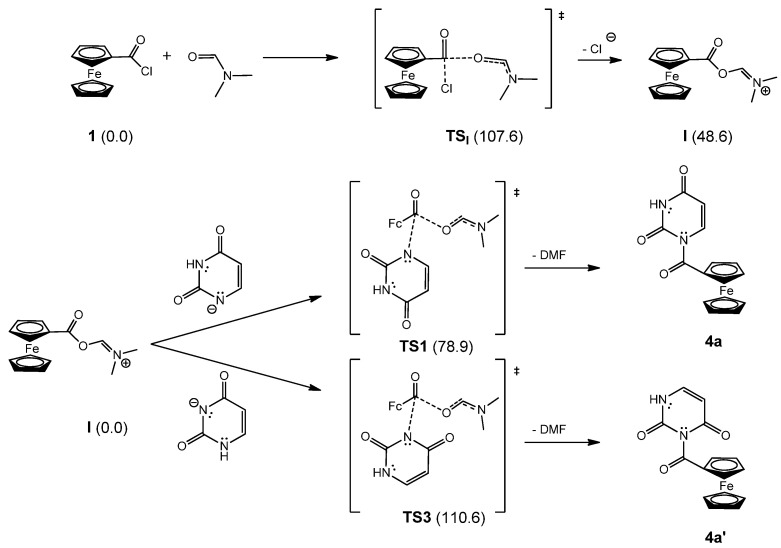
Proposed mechanism of the regioselective ferrocenoylation of uracil observed in DMF. All relative energies (ΔG, in kJ/mol; in parentheses) are calculated at the SMD-B3LYP/6-31G(d)/SDD level of theory in *N*,*N*-dimethylformamide (ε = 37.22) as a model solvent. ‡ = symbol for a transition state.

To rationalise the mechanism underlying the regioselective reaction in DMF, quantum chemical calculations were performed. It is known that DMF can act as an oxygen nucleophile [35]. We have previously observed similar reactivity with DMSO, which acts as an oxygen nucleophile in the analogous chemistry of purine–ferrocenoyl conjugates [30,36]. In our case, DMF undergoes an S_N_2-like reaction with ferrocenoyl chloride (**1**) (Scheme 2). The calculated energy barrier for this reaction is only 107.6 kJ/mol, which can be easily overcome at slightly elevated temperatures or even at room temperature. The corresponding transition state structure **TS_I_** is characterised by simultaneous O–C bond formation and C–Cl bond breaking (Scheme 2). The reaction is endergonic by 48.6 kJ/mol, resulting in the formation of the reactive intermediate [FcCOOCHN(Me)_2_]^+^ (**I**).

This cationic form **I** is susceptible to nucleophilic attack by the uracil anion, deprotonated at either the *N*1 or *N*3 position. Thus, in the second step, the reactive intermediate **I** undergoes a reaction in which an N–C bond is formed, followed by the elimination of a DMF molecule. The transition state structure **TS1**, which connects the *N*1 uracil anion and its ferrocenoylated form **4a**, is more than 30 kJ/mol more stable than the corresponding transition state structure **TS3** for the competing process at the *N*3 position of the uracil anion. As shown earlier, no significant energy difference between the two products **4a** and **4a’** was calculated, supporting the claim that regioselectivity is governed by the kinetic profile of the two reactions.

A similar observation was reported for reactions between pyrimidine base anions (at the *N*1 or *N*3 position) and a series of acyl chlorides: the regioselectivity in the ferrocenoylation of nucleobases was kinetically controlled [29]. Here, we demonstrate that the solvent can influence the observed regioselectivity by forming a short-lived intermediate that determines the preference for the *N*1-reaction. Unlike DMF, CH_3_CN cannot produce a comparable regioselective outcome. Our computational results suggest that the reaction between FcCOCl and CH_3_CN does not lead to the formation of the intermediate required for regioselective differentiation between the two pathways. All attempts to optimise the expected intermediate resulted in separated reactants. Acetonitrile is a weak nucleophile and must be activated by metal complexes to enhance its reactivity [37]. This explains why the ferrocenoylation of uracil in acetonitrile yields a reaction mixture containing both *N*1- and *N*1/*N*3-products.

### 2.2. Regioselectivity of the C5 Coupling Reaction of Uracil Derivatives

In the continuation of the research, model reaction conditions were applied to the *C*5 derivatives of uracil to examine the regioselectivity of these reactions. The groups attached to the *C*5 position of uracil can be electron-withdrawing or electron-donating, which may affect the course and regioselectivity of the reactions. Two reactions were carried out using ferrocenoyl chloride (**1**) as the acylating agent, referred to as method A and method B (Table 2). Method A used NaH as the base, with reactions performed in DMF, while method B used Et_3_N as the base and CH_3_CN as the solvent. The reactions demonstrated the regiospecificity of method A for uracil and its *C*5 derivatives. In all tested reactions, only the *N*1 product (**4a**–**f**) was formed (Table 2, rows 1–6), as confirmed by NMR studies.

In the reactions carried out with Et_3_N as the base in CH_3_CN (method B), two products were isolated in all cases: *N*1 products **4a–f**, obtained as red crystals in yields of 23–59%, and *N*1/*N*3 products **6a–f**, obtained as dark red crystals in yields of 22–35% (Table 2, lines 7–12). The results show that when an electron-donating group (CH_3_) is attached at the *C*5 position, the proportion of the *N*1 isomer is significantly higher than that of *N*1/*N*3 isomers (74:26, Table 2, line 8). However, when an electron-accepting group is located at the *C*5 position, the ratio changes, and both isomers are formed in approximately similar proportions (Table 2, lines 9–12). It can also be concluded that the electronegativity and size of the halogen atoms at the *C*5 position do not have a major influence on the regioselectivity of these reactions (Table 2).

The reaction of *N*-ferrocenoylation with 6-methyluracil (**5**) was attempted using both methods A and B, but no reaction occurred; neither *N*1 nor *N*1/*N*3 products were formed. The lack of reaction can primarily be attributed to the steric effect of the electron-donating group at the *C*6 position.

### 2.3. Biological Evaluation of Ferrocene–Pyrimidine Conjugates

#### 2.3.1. Acellular ROS Activity Testing

The ROS-inducing activity of the compounds measures their ability to increase the formation of reactive oxygen species (ROS), which can damage biomolecules and cellular structures, leading to cytotoxicity and cell death. A commonly used method for detecting ROS is the acellular fluorimetric measurement of the fluorescence intensity of the dye DCFH_2_-DA. This dye is initially non-fluorescent; it is deacetylated to DCFH_2_, which is then oxidised by ROS to form the fluorescent products DCF (2,7-dichlorofluorescein) and fluorescein. For acellular measurements, the diacetate form (DCFH_2_-DA) is chemically hydrolysed using NaOH and then added at specific concentrations to the tested compounds. DCFH_2_-DA can detect hydroxyl and peroxyl radicals, as well as hypochlorous acid and peroxynitrite—and, to a lesser extent, superoxide radical and hydrogen peroxide [38]. An increase in fluorescence intensity over time and with increasing concentration indicates enhanced ROS formation.

**Figure 6:**
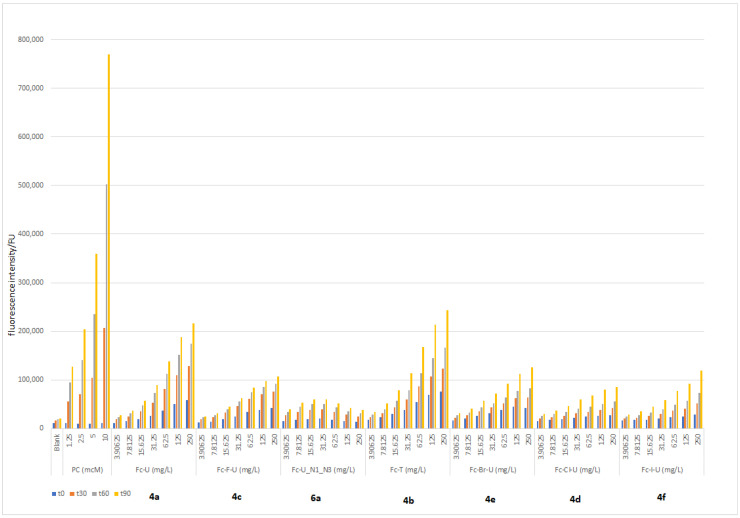
Acellular ROS activity for synthesised compounds and positive control, 6-methylpurine, with DCFH_2_-DA assay. Intensity of fluorescence increases with concentration of analysed substance as well as with time.

The results show that the fluorescence intensity increases over time, reaching a maximum at *t* = 90 min, and also increases with the analyte concentration (Figure 6). Compounds **4a** (Fc-U) and **4b** (Fc-T) showed a significant increase in fluorescence intensity. This ROS activity suggests the potential biological activity of these compounds as cytotoxic agents against tumour cell lines. In addition to their ROS-inducing capacity, the ferrocenoylated uracil derivatives were further evaluated using a fluorescence quenching assay (Figure S1), which revealed their ability to quench fluorescence. All compounds exhibit concentration-dependent fluorescence quenching. Although the differences in fluorescence quenching among the compounds are relatively modest, the observed activity confirms the presence of redox interactions, which align with their previously demonstrated oxidative activity.

#### 2.3.2. Testing the Immunostimulatory Activity In Vivo

Our aim was to determine whether these compounds could stimulate or modulate the immune response in a living organism, which is critical for potential applications in antiviral or anticancer therapies. Tumour development and survival are regulated in a complex and often chaotic manner, involving interactions between cancer cells, normal stromal cells, and the host immune system [39]. However, tumours have evolved multiple mechanisms to evade immune surveillance and destruction. The identification and development of compounds that can upregulate the immune response holds great promise for enhancing cancer treatment and offers new opportunities in cancer therapy. The synthesis and various biological activities of ferrocene–pyrimidine conjugates have been described to date [40,41,42].

**Figure 7:**
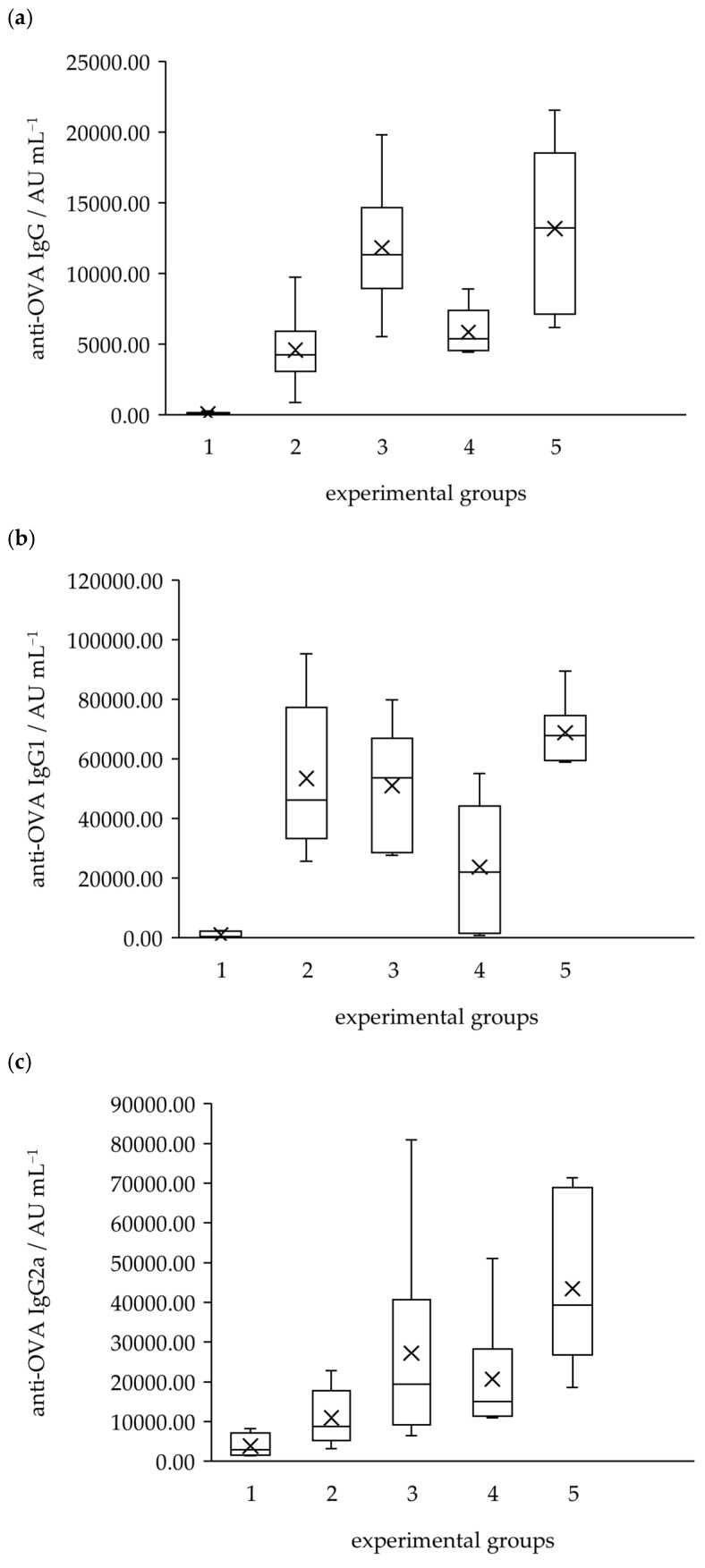
The effect of the tested compounds on the production of total anti-OVA IgGs as well as anti-OVA IgG1 and anti-OVA IgG2a in the sera of NIH/OlaHsd female mice (*n* = 6) after the second booster. Total anti-OVA IgG (**a**); anti-OVA IgG1 (**b**); and anti-OVA IgG2a (**c**). Experimental groups were as follows: 1. OVA; 2. OVA + MDP, 3. OVA + Fc-U (**4a**), 4. OVA + Fc-F-U (**4c**), and 5. OVA + Fc-T (**4b**). x denotes group mean value.

Based on the results of ROS activity measurements, the influence of Fc-U (**4a**), Fc-T (**4b**), and Fc-F-U (**4c**) on specific immune responses was investigated. Immunostimulatory activity was assessed in a mouse model to determine whether the synthesised compounds affect antigen-specific immune reactions (Figure 7a). Specifically, the ferrocene–pyrimidine conjugates were evaluated for their effects on the immune response against the model antigen ovalbumin (OVA). NIH/OlaHsd mice were immunised with OVA mixed with the test compounds. To assess the nature of the induced immune response in terms of Th1/Th2 polarisation, levels of Th1-associated IgG2a and Th2-associated IgG1 antibody isotypes were measured (Figure 7b,c). The results revealed a significant increase in IgG production in the sera of mice immunised with OVA mixed with compounds **4a**, **4b**, and **4c**. Muramyl dipeptide (MDP) was used as a positive control. As expected, MDP triggered a predominantly Th2-oriented response, with a significant increase in IgG1 and only a marginal increase in IgG2a compared to the control group immunised with OVA in saline. Compounds **4a** and **4b** enhanced the humoral immune response and promoted the production of total anti-OVA antibodies to a greater extent than MDP. The production of IgG2a antibodies is typically associated with a Th1-type immune response, which is important for promoting cell-mediated immunity and is often linked to antitumor and antiviral activity. In this study, the observed significant increase in IgG2a levels in mice immunised with **4a** and **4b** conjugates suggests that these compounds may skew the immune response towards a Th1 profile. This indicates their relevance in therapeutic strategies aimed at enhancing cellular immune responses, such as cancer immunotherapy. Preliminary results from the OVA-specific IgG analysis demonstrated that the synthesised ferrocene–pyrimidine conjugates modulate the OVA-specific immune response and alter the IgG1/IgG2a ratio. Taken together with their ROS-inducing activity, these findings support the further investigation of these compounds for potential application in antitumor therapy.

## 3. Materials and Methods

### 3.1. General

The syntheses were carried out under argon atmosphere in dry solvents. Thin layer chromatography was performed on pre-coated Merck silica gel 60F-254 plates (Darmstadt, Germany) using an appropriate solvent system to monitor reaction progress and for comparison purposes, and spots were detected under UV light (254 nm). The products were purified by column chromatography (Fluka, Geneva, Switzerland, silica gel, 90 Å, 70-230 mesh) using CH_2_Cl_2_ and CH_2_Cl_2_/(CH_3_)_2_CO mixtures. The melting points were determined using a Buechi apparatus (Uster, Switzerland). IR spectra were recorded for CH_2_Cl_2_ solutions or KBr pastilles using a Bruker ALPHA-FT-IR spectrophotometer (Billerica, MA, USA). The standard ^1^H, ^13^C{^1^H} NMR spectra of DMSO-*d*_6_ and CDCl_3_ solutions were recorded with a Varian INOVA 400 spectrometer (Palo Alto, CA, USA). The spectrometer operated at 399.6 MHz (^1^H) and 100.5 MHz (^13^C). The chemical shifts in the ^1^H NMR and ^13^C NMR spectra were expressed in parts per million (ppm) and related to the residual resonances of the solvent. Elemental analysis was performed on a PerkinElmer 2400 Series II CHNS Analyzer (Waltham, MA, USA); the main standard is acetanilide.

### 3.2. Materials

All pyrimidine derivatives [R(*C*5) = H, CH_3_, F, Cl, Br, I], [R(*C*5) = H, R′(*C*6) = CH_3_] were purchased from Sigma-Aldrich (St. Louis, MO, USA).

### 3.3. Preparation of Substrates

Ferrocenoyl chloride (FcCOCl, **1**) [29], ferrocenoyl ethyl carbonate (FcCOOCOOEt, **2**) [29], and ferrocenoyl benzotriazole (FcCOOBt, **3**) [31] were synthesised according to known procedure. The synthesis and spectral characterisation of compounds **4a–c** were described in an earlier paper [29].

### 3.4. Optimisation of the Reaction Conditions for the N-Ferrocenoylation of Uracil for Table 1, Tables S1 and S2

Deprotonating agent (NaH or K_2_CO_3_ or TEA, 1.5 mmol) was added in portions to uracil (1 mmol) suspended in solvent (DMF, CH_3_CN or 1,4-dioxane, 3 mL). After stirring for 30–90 min at room temperature, FcCOCl (**1**) (1.2 mmol) or FcCOOCOOEt (**2**) or FcCOOBt (**3**) was added dropwise to the clear solution. The mixture was stirred for 30 min, then neutralised with a 10% aqueous solution of citric acid and extracted with CH_2_Cl_2_. The organic layer was washed with water, and the solvents were evaporated in vacuo. Subsequent purification by column chromatography or preparative thin-layer chromatography yielded *N*1- or/and *N*1/*N*3-ferrocenoylated pyrimidine bases as separate fractions (Table 1, Tables S1 and S2).

### 3.5. General Procedure for the Preparation of N1- or/and N1/N3-Ferrocenoylated C5 Derivatives of Pyrimidine Bases for Table 2

Method A: *C*5-substituted uracil derivatives (1 mmol) were suspended in 1 mL DMF, and NaH (1.5 mmol) was added with constant stirring for 90 min at room temperature. Subsequently, the previously prepared ferrocenoyl chloride (**1**) (1.2 mmol) was added to the reaction mixture. After stirring for 30 min at room temperature, the reaction mixture was neutralised by adding 10% citric acid solution and extracted with CH_2_Cl_2_. The organic layer was then washed with water until it was neutral, dried with Na_2_SO_4_ and evaporated to dryness on a rotary evaporator. The crude product was dissolved in CH_2_Cl_2_ and purified by column chromatography on silica gel with the eluent CH_2_Cl_2_/CH_3_COCH_3_ 10:0.2. The column chromatography yielded a product, the red *N*1-regioisomer. The purified product is purified again by chromatography on a silica gel plate with the eluent CH_2_Cl_2_/CH_3_COCH_3_ 10:0.1 (Table 2).

Method B: *C*5-substituted uracil derivatives (1 mmol) were suspended in 3 mL CH_3_CN, and (C_2_H_5_)_3_N (1.5 mmol) was added. The mixture was stirred constantly at room temperature for 90 min. Subsequently, the previously prepared ferrocenoyl chloride (**1**) (2.4 mmol) was added to the reaction mixture. After stirring for 30 min at room temperature, the reaction mixture was neutralised by adding 10% citric acid solution and extracted with CH_2_Cl_2_. The organic layer was then washed with water until neutral, dried with Na_2_SO_4_, and evaporated to dryness on rotary vapour. The crude product was dissolved in CH_2_Cl_2_ and purified by column chromatography on silica gel with the eluent CH_2_Cl_2_/CH_3_COCH_3_ 10:0.2. Two products were obtained by column chromatography: *N*1- and *N*1/*N*3-products. The purified products are purified again, each separately, by chromatography on a silica gel plate in the system CH_2_Cl_2_/CH_3_COCH_3_ 10:0.2 (Table 2).

### 3.6. Characterisation of Products

*N*1/*N*3-diferrocenoyl uracil (**6a**): Red crystals, m.p. > 200 °C. TLC analysis (SiO_2_, CH_2_Cl_2_/acetone 10:1) showed *R*_f_ = 0.70. ^1^H NMR (400 MHz, CDCl_3_, 25 °C): δ = 7.68 (d, *J*_H,H_ = 8.1 Hz, 1H; C6-H), 5.92 (d, *J*_H,H_ = 8.2 Hz, 1H; C5-H), 4.89 (s, 2H; CH-Fc), 4.77 (s, 2H; CH-Fc), 4.68 (s, 2H; CH-Fc), 4.63 (s, 2H; CH-Fc), 4.38 (s, 5H; Cp-H), and 4.29 (s, 5H; Cp-H) ppm. ^13^C NMR (100 MHz, CDCl_3_, 25 °C): δ = 172.3 (FcCO), 161.5 (C4), 147.8 (C2), 139.1 (C6), 102.9 (C5), 73.9 (CH-Fc), 73.7 (CH-Fc), 73.5 (CH-Fc), 73.4 (CH-Fc), 71.9 (Cq-Fc), 71.0 (Cp), and 70.9 (Cp) ppm. IR (CH_2_Cl_2_): ῦ_max_ = 3055 (w, CH, Fc), 1693 (s, C=O), 1438 (w, purine ring breathing), and 1266 (m, C–N) cm^−1^. Elemental analysis calcd (%) for C_26_H_20_Fe_2_N_2_O_4_: C 58.24, H 3.76, and N 5.23; found: C 58.44, H 3.75, and N 5.37.

*N*1/*N*3-diferrocenoyl thymine (**6b**): Red-orange crystals m.p. > 200 °C. TLC analysis (SiO_2_, CH_2_Cl_2_/acetone 10:0.2) showed *R*_f_ = 0.75. ^1^H NMR (400 MHz, CDCl_3_, 25 °C): δ = 7.53 (s, 1H; C6-H), 4.88 (pt, 2H; CH-Fc), 4.76 (pt, 2H; CH-Fc), 4.65 (t, 2H; CH-Fc), 4.37 (s, 5H; Cp-H), 4.28 (s, 5H; Cp-H), and 2.04 (d, 3H; CH3) ppm. ^13^C NMR (100 MHz, CDCl_3_, 25 °C): δ = 172.6 (FcCO), 172.6 (FcCO), 161.7 (C4), 148.0 (C2), 134.7 (C6), 111.5 (C5), 73.6 (CH-Fc), 73.3 (CH-Fc), 72.5 (CH-Fc), 72.3 (CH-Fc), 72.0 (Cq-Fc), 70.9 (Cp), 70.9 (Cp), and 12.7 (CH_3_) ppm. IR (CH_2_Cl_2_): ῦ_max_ = 3082 (w, CH, Fc), 2943 (w, CH, Fc), 1688 (s, C=O), 1417 (s, purine ring breathing), and 1263 (C-CH_3_, ring) cm^−1^. Elemental analysis calcd (%) for C_27_H_22_Fe_2_N_2_O_4_: C 58.94, H 4.03, and N 5.09; found: C 55.61, H 3.75, and N 8.67.

*N*1/*N*3-diferrocenoyl-5-F-uracil (**6c**): Red crystals, m.p. > 200 °C. TLC analysis (SiO_2_, CH_2_Cl_2_/acetone 10:0.2) showed *R*_f_ = 0.71. ^1^H NMR (400 MHz, CDCl3, 25 °C): δ = 7.81 (d, *J*_H,H_ = 8.1 Hz, 1H; C6-H), 4.89 (s, 2H; CH-Fc), 4.78 (s, 2H; CH-Fc), 4.67 (s, 4H; CH-Fc), 4.39 (s, 5H, Cp-H), and 4.29 (s, 5H, Cp-H) ppm. ^13^C NMR (400 MHz, CDCl3, 25 °C): δ = 171.0 (FcCO), 170.5 (FcCO), 155.2 (C4), 146.1 (C2), 140.6 (C6), 122.9 (C5), 73.4 (CH-Fc), 73.3 (CH-Fc), 72.6 (CH-Fc), 71.3 (Cp), and 70.6 (Cp) ppm. IR (CH_2_Cl_2_): ῦ_max_ = 3354 (w, NH), 2960 (w, CH, Fc), 1680 (s, C=O), and 1474 (s, purine ring breathing) cm^−1^. Elemental analysis calcd (%) for C_26_H_19_Fe_2_N_2_O_4_F: C 56.35, H 3.46, and N 5.06; found: C 56.36, H 3.49, and N 5.10.

*N*1-ferrocenoyl-5-Cl-uracil (**4d**): Red crystals m.p. > 200 °C. TLC analysis (SiO_2_, CH_2_Cl_2_/acetone 10:0.2) showed *R*_f_ = 0.20. ^1^H NMR (400 MHz, DMSO-*d*_6_, 25 °C): δ = 11.99 (s, 1H; N3-H), 8.46 (s, 1H; C6-H), 4.89 (s, 2H; CH-Fc), 4.74 (s, 2H; CH-Fc), and 4.35 (s, 5H, Cp-H) ppm. ^13^C NMR (100 MHz, DMSO-*d*_6_, 25 °C): δ = 171.9 (FcCO), 159.3 (C4), 148.6 (C2), 137.6 (C6), 108.7 (C5), 73.6 (CH-Fc), 71.5 (CH-Fc), 72.0 (Cq-Fc), and 70.7 (Cp) ppm. IR (KBr): ῦ_max_ = 3464 (w, NH), 3158 (w, CH, Fc), 1698 (s, C=O), 1426 (s, purine ring breathing), and 863 (C–Cl) cm^−1^. Elemental analysis calcd (%) for C_15_H_10_FeN_2_O_3_Cl: C 50.39, H 2.82, and N 7.84; found: C 50.28, H 2.71, and N 7.77.

*N*1/*N*3-diferrocenoyl-5-Cl-uracil (**6d**): Red crystals m.p. > 200 °C. TLC analysis (SiO_2_, CH_2_Cl_2_/acetone 10:0.2) showed *R*_f_ = 0.73. ^1^H NMR (400 MHz, CDCl3, 25 °C): δ = 7.91 (s, 1H; C6-H), 4.88–4.86 (m, 2H; CH-Fc), 4.77 (s, 2H; CH-Fc), 4.70 (s, 2H; CH-Fc), 4.67–4.65 (m, 2H; CH-Fc), 4.38 (s, 5H; Cp-H), and 4.30 (s, 5H; Cp-H) ppm. ^13^C NMR (100 MHz, CDCl_3_, 25 °C): δ = 171.3 (FcCO), 171.2 (FcCO), 157.6 (C4), 147.1 (C2), 135.9 (C6), 110.2 (C5), 74.1 (CH-Fc), 73.7 (CH-Fc), 73.1 (CH-Fc), 72.7 (Cq-Fc), 72.0 (CH-Fc), 71.1 (Cp), and 71.1 (Cp) ppm. IR (CH_2_Cl_2_): ῦ_max_ = 3052 (w, CH, Fc), 1699 (s, C=O), 1474 (s, purine ring breathing), and 863 (C–Cl) cm^−1^. Elemental analysis calcd (%) for C_26_H_19_Fe_2_N_2_O_4_Cl: C 58.35, H 3.58, and N 5.24; found: C 58.61, H 3.45, and N 5.17.

*N*1-ferrocenoyl-5-Br-uracil (**4e**): Red crystals m.p. > 200 °C. TLC analysis (SiO_2_, CH_2_Cl_2_/acetone 10:0.2) showed *R*_f_ = 0.22. ^1^H NMR (400 MHz, DMSO-*d*_6_, 25 °C): δ = 11.60 (s, 1H; N3-H), 8.40 (s, 1H; C6-H), 4.87 (s, 2H; CH-Fc), 4.73 (s, 2H; CH-Fc), and 4.34 (s, 5H; Cp-H) ppm. ^13^C NMR (100 MHz, DMSO-*d*_6_, 25 °C): δ = 172.2 (FcCO), 159.8 (C4), 149.2 (C2), 140.2 (C6), 97.9 (C5), 74.1 (CH-Fc), 73.4 (CH-Fc), 72.6 (Cq-Fc), 72.0 (CH-Fc), and 71.2 (Cp) ppm. IR (KBr): ῦ_max_ = 3406 (w, NH), 3168 (w, CH, Fc), 2848 (w, CH), 1699 (s, C=O), and 1421 (s, purine ring breathing) cm^−1^. Elemental analysis calcd (%) for C_15_H_10_FeN_2_O_3_Br: C 44.81, H 2.51, and N 6.97; found: C 44.86, H 2.58, and N 6.91.

*N*1/*N*3-diferrocenoyl-5-Br-uracil (**6e**): Dark red crystals m.p. > 200 °C. TLC analysis (SiO_2_, CH_2_Cl_2_/acetone 10:0.2) showed *R*_f_ = 0.73. ^1^H NMR (400 MHz, CDCl_3_, 25 °C): δ = 8.03 (s, 1H; C6-H), 4.87 (t, 2H; CH-Fc), 4.77 (s, 2H; CH-Fc), 4.70 (s, 2H; CH-Fc), 4.65 (pt, 2H; CH-Fc), 4.38 (s, 5H; Cp-H), and 4.30 (s, 5H; Cp-H) ppm. ^13^C NMR (100 MHz, CDCl_3_, 25 °C): δ = 171.3 (FcCO), 171.2 (FcCO), 157.5 (C4), 147.3 (C2), 138.3 (C6), 98.1 (C5), 74.1 (CH-Fc), 73.6 (CH-Fc), 73.1 (Cq-Fc), 72.0 (CH-Fc), 71.1 (Cp), and 71.1 (Cp) ppm. IR (CH_2_Cl_2_): ῦ_max_ = 3046 (w, CH, Fc), 1699 (s, C=O), and 1421 (s, purine ring breathing) cm^−1^. Elemental analysis calcd (%) for C_26_H_19_Fe_2_N_2_O_4_Br: C 50.77, H 3.11, and N 4.55; found: C 50.71, H 3.09, and N 54.61.

*N*1-ferrocenoyl-5-I-uracil (**4f**): Red crystals m.p. > 200 °C. TLC analysis (SiO_2_, CH_2_Cl_2_/acetone 10:0.2) showed *R*_f_ = 0.27. ^1^H NMR (400 MHz, DMSO-*d*_6_, 25 °C): δ = 11.75 (s, 1H; N3-H), 8.46 (s, 1H; C6-H), 4.88 (t, 2H; CH-Fc), 44.76 (t, 2H; CH-Fc), and 4.37 (s, 5H; Cp-H) ppm. ^13^C NMR (100 MHz, DMSO-*d*_6_, 25 °C): δ = 172.47 (FcCO), 161.3 (C4), 149.6 (C2), 144.9 (C6), 74.1 (CH-Fc), 72.5 (Cq-Fc), 72.0 (CH-Fc), 71.8 (C5), and 71.2 (Cp) ppm. IR (KBr): ῦ_max_ = 3286 (w, NH), 3082 (w, CH, Fc), 1691 (s, C=O), and 1396 (s, purine ring breathing) cm^−1^. Elemental analysis calcd (%) for C_15_H_12_FeN_2_O_3_I: C 40.12, H 2.24, and N 6.23; found: C 40.01, H 2.29, and N 6.18.

*N*1/*N*3-diferrocenoyl-5-I-uracil (**6f**): Red crystals m.p. > 200 °C. TLC analysis (SiO_2_, CH_2_Cl_2_/acetone 10:0.2) showed *R*_f_ = 0.71. ^1^H NMR (400 MHz, CDCl_3_, 25 °C): δ = 8.13 (s, 1H; C6-H), 4.88 (s, 2H; CH-Fc), 4.76 (s, 2H; CH-Fc), 4.71 (s, 2H; CH-Fc), 4.65 (m, 2H; CH-Fc), 4.38 (s, 5H; Cp-H), and 4.31 (s, 5H; Cp-H) ppm. ^13^C NMR (100 MHz, CDCl_3_, 25 °C): δ = 171.1 (FcCO), 170.7 (FcCO), 157.9 (C4), 147.1 (C2), 143.0 (C6), 73.7 (CH-Fc), 73.2 (CH-Fc), 72.6 (Cq-Fc), 71.5 (CH-Fc), 70.7 (Cp), 70.6 (Cp), and 69.3 (C5) ppm. IR (CH_2_Cl_2_): ῦ_max_ = 3063 (w, CH, Fc), 1713, 1682 (s, C=O), and 1444 (s, purine ring breathing) cm^−1^. Elemental analysis calcd (%) for C_26_H_19_Fe_2_N_2_O_4_I: C 47.17, H 2.89, and N 4.23; found: C 55.61, H 2.75, and N 4.28.

### 3.7. Density Functional Theory (DFT) Calculations

Geometries were optimised at the B3LYP level of theory, as implemented in the Gaussian 16 software package. The basis set for optimisation was standard Pople’s 6-31G(d) on non-metal atom centres, and the Stuttgart–Dresden–Bonn (SDD) basis set with effective core potential (ECP) was used for Fe. This DFT level has proven quite successful for transition-metal compounds and is well suited for the description of structures, energies, vibrational frequencies, and other properties. Harmonic frequencies were computed from analytical second derivatives. Gibbs energies of solvation were determined by using the SMD continuum solvation model at the same level of theory. The solvent relative permittivity of ε = 37.22 [*N*,*N*-dimethylformamide (DMF)] was used.

IRC calculations (intrinsic reaction coordinate, as implemented in Gaussian 16) [43] were performed to identify the minima connected through the transition state. The initial geometries used were that of the corresponding transition structures, and the paths were followed in both directions from that point. This method verified that a given transition state structure indeed connected the presumed energy minimum structures.

### 3.8. In Vivo Evaluation of Immunostimulatory Activity

Bovine serum albumin, Tween 20, monoclonal anti-chicken egg albumin (clone OVA-14 mouse IgG1 isotype), o-phenylenediamine dihydrochloride, and MDP were provided by Sigma-Aldrich, Schnelldorf, Germany. Horseradish peroxidase-conjugated goat anti-mouse IgG (HRP-anti-mouse IgG) was from Bio-Rad Laboratories (Hercules, CA, USA). Biotin-conjugated rat anti-mouse IgG1 and anti-mouse IgG2a monoclonal antibodies and streptavidin–peroxidase were from PharMingen, Becton Dickinson (San Diego, CA, USA). Chemicals for buffers and solutions were purchased from Kemika d.d. (Zagreb, Croatia). Ovalbumin was purchased from Serva Electrophoresis GmbH, Heidelberg, Germany. Details can be found in the Supplementary Materials.

## 4. Conclusions

The *N*-ferrocenoylation of uracil demonstrated that regioselectivity depends strongly on the choice of solvent. Reactions in DMF were regiospecific, yielding only the *N*1 product under all conditions tested, whereas reactions in CH_3_CN produced both *N*1 and *N*1/*N*3 products, with the relative proportions strongly influenced by the base and the duration of nucleobase salt formation. Computational studies confirmed that, in DMF, a short-lived reactive intermediate is stabilised, favouring nucleophilic attack at the *N*1 position, while in CH_3_CN, such an intermediate does not form, resulting in a mixture of regioisomers. Applying the optimised conditions to *C*5-substituted uracil derivatives showed that electron-donating groups increase the proportion of the *N*1-isomer, while electron-accepting groups result in more balanced *N*1 and *N*1/*N*3 ratios. Halogen substitution at the *C*5 position had minimal effect on regioselectivity. The synthesised ferrocenoylated uracil derivatives exhibited time- and concentration-dependent ROS generation, measurable fluorescence quenching, and immunomodulatory activity, indicating that their biological activity is closely linked to their redox properties. These findings highlight the importance of solvent and substituent effects in controlling regioselectivity, as well as the potential of ferrocene–pyrimidine conjugates as redox-active therapeutic agents, including applications in anticancer and immunomodulatory strategies.

## Figures and Tables

**Table 1:** Regioselectivity of reactions using FcCOCl (**1**).

Lines	Base	Solvent	Reaction Time/min	4a η (%)	6a η (%)	4a:6a
1 2 3	NaH K_2_CO_3_ Et_3_N	DMF	30	65 45 30	- - -	
4 5 6	NaH K_2_CO_3_ Et_3_N		60	72 49 24	- - -	
7 8 9	NaH K_2_CO_3_ Et_3_N		90	74 68 52	- - -	
10 11 12	NaH K_2_CO_3_ Et_3_N	CH_3_CN	30	41 5 20	- - 3	- - 87:13
13 14 15	NaH K_2_CO_3_ Et_3_N		60	60 17 19	4 3 14	94:6 85:15 62:38
16 17 18	NaH K_2_CO_3_ Et_3_N		90	45 62 42	7 3 27	87:13 96:4 60:40
19 20 21	NaH K_2_CO_3_ Et_3_N	1,4-dioxane	60	- - 17	- - 4	- - 83:17
22	Et_3_N		90	15	3	84:16

**Table 2:** Experimentally obtained reaction yield.

Lines		Product	η (%)	Product	η (%)	4:6
1	Method A	**4a**	74	**6a**	-	-
2		**4b**	41	**6b**	-	-
3		**4c**	75	**6c**	-	-
4		**4d**	76	**6d**	-	-
5		**4e**	68	**6e**	-	-
6		**4f**	65	**6f**	-	-
7	Method B	**4a**	45	**6a**	33	58:42
8		**4b**	59	**6b**	22	74:26
9		**4c**	48	**6c**	32	59:41
10		**4d**	23	**6d**	35	50:50
11		**4e**	35	**6e**	30	52:48
12		**4f**	34	**6f**	32	54:46

## Data Availability

The original contributions presented in this study are included in the article. Further inquiries can be directed to the corresponding author.

## Supplementary Materials

The following supporting information can be downloaded at: https://www.mdpi.com/article/10.3390/molecules31061054/s1. 1. Table S1. Regioselectivity of reactions using FcCOOCOOEt (**2**); Table S2. Regioselectivity of reactions using FcCOOBt (**3**); 2. ELISA for qualitative and quantitative determination of anti-OVA IgG; 3. Acellular ROS activity testing [including Figure S1. Fluorescence quenching test using a 0.1 μM F-DA (fluorescein-diacetate solution)]; 4. IR and NMR Spectra; 5. XYZ Coordinates of optimised stationary points. Figure S1. Fluorescence quenching test using a 0.1 μM F-DA (fluorescein-diacetate solution). Results are shown for the tested compounds at concentrations of 62.5, 125, and 250 mgL^−1^; Figure S2. (a) ^1^H NMR and (b) ^13^C APT NMR of compd. **4d**; Figure S3. (a) ^1^H NMR and (b) ^13^C NMR of compd. **4e**; Figure S4. (a) ^1^H NMR and (b) ^13^C APT NMR of compd. **4f**; Figure S5. (a) ^1^H NMR and (b) ^13^C NMR of compd. **6a**; Figure S6. (a) ^1^H NMR and (b) ^13^C NMR of compd. **6b**; Figure S7. (a) ^1^H NMR and (b) ^13^C APT NMR of compd. **6c**; Figure S8. (a) ^1^H NMR and (b) ^13^C NMR of compd. **6d**; Figure S9. (a) ^1^H NMR and (b) ^13^C NMR of compd. **6e**; Figure S10. (a) ^1^H NMR and (b) ^13^C APT NMR of compd. **6f**; Figure S11. IR spectra of compd. **4a** i **6a**; Figure S12. IR spectra of compd. **4b** i **6b**; Figure S13. IR spectra of compd. **4c** i **6c**; Figure S14. IR spectra of compd. **4d** i **6d**; Figure S15. IR spectra of compd. **4e** i **6e**; Figure S16. IR spectra of compd. **4f** i **6f** [44,45].
