# Supplementary material for: Ferrocenoylation of Uracil Derivatives: Study of Reaction Regioselectivity and Biological Activity
Source: Molecules. 2026 Mar 23;31(6):1054. doi: 10.3390/molecules31061054 (PMC13028637; doi:10.3390/molecules31061054)
Supplement: Supplementary file 1 [file molecules-31-01054-s001.zip › molecules-4179066-supplementary.pdf]

Supplementary Materials

Ferrocenoylation of Uracil Derivatives: Study of Reaction Regioselectivity and Biological Activity

Jasmina Lapić[1], Ivana Kuzman[1], Ruža Frkanec[2], Leo Frkanec[3], Senka Djaković[1,*]

[1] Faculty of Food Technology and Biotechnology, University of Zagreb, Pierottijeva 6, 10000 Zagreb, Croatia; jasmina.lapic@pbf.unizg.hr (J.L.)
[2] Centre for Research and Knowledge Transfer in Biotechnology, University of Zagreb, Rockefellerova 10, 10000 Zagreb, Croatia; ruza.frkanec@unizg.hr
[3] Rudjer Bošković Institute, Bijenička 54, 10000 Zagreb, Croatia; frkanec@irb.hr

* Correspondence: Senka Djaković, senka.djakovic@pbf.unizg.hr

Table of contents

1. Table S1 and S2
2. ELISA for qualitative and quantitative determination of anti-OVA IgG
2. Acellular ROS activity testing
3. NMR and IR Spectra
4. XYZ Coordinates of optimized stationary points

1. Table S1 and S2

Table S1. Regioselectivity of reactions using FcCOOCOOEt (**2**)

Base	Solvent	Deprotonation time/ min	Product 4a η (%)	Product 6a η (%)
NaH	DMF	60	7.8	-
K_2CO_3			3.0	-
$(C_2H_5)_3N$			4.0	-
NaH	CH_3CN	60	-	-
K_2CO_3			4.0	-
$(C_2H_5)_3N$			3.2	-

Table S2. Regioselectivity of reactions using FcCOOBt (**3**)

Base	Solvent	Deprotonation time/ min	Product 4a η (%)	Product 6a η (%)
NaH	DMF	30	14	-
K_2CO_3			17	-
$(C_2H_5)_3N$			-	-
NaH	CH_3CN	30	-	-
K_2CO_3			-	-
$(C_2H_5)_3N$			-	-

NaH	DMF	60	31	-
K_2CO_3			22	-
$(C_2H_5)_3N$			-	-
NaH	CH_3CN	60	4.7	-
K_2CO_3			-	-
$(C_2H_5)_3N$			-	-

2. ELISA for qualitative and quantitative determination of anti-OVA IgG

Anti-OVA antibodies were determined by previously described ELISA modified as follows [44,45]. Briefly, flat-bottomed high binding ELISA plates (Costar, USA) were coated with 100 μL of 15 μgmL^{-1} OVA solution in carbonate buffer, pH 9.2, overnight at room temperature (RT). Non-specific antibody binding was blocked by incubation with 0.5 % (w/v) BSA in PBS–T (0.05 % (v/v) Tween 20 in PBS) buffer (200 μL per well) for 2 h at 37 ºC. Standard antibody preparation and mouse sera to be tested (100 μL per well) were added in serial two-fold dilutions and incubated overnight at RT. All samples were analysed in duplicates. Plates were washed and 100 μL of goat HRP–anti-mouse IgG (4 000 × diluted) was added and incubated 2 h at 37 ºC. After washing, the substrate solution consisting of 0,6 mgmL^{-1} OPD solution in citrate–phosphate buffer, pH 5.0, with 0.5 μL 30 % H_2O_2 /mL was added (100 μL per well) and incubated for 30 min at RT in the dark. The enzymatic reaction was stopped with 50 μL per well 12.5 % H_2SO_4 and absorbency at 492 nm was measured using a microplate reader (Reader 530; Organon Teknika, The Netherlands). All washings (three times after each step) were done with PBS–T buffer in microplate washer (Multiwash; Labsystems, Finland).

Quantitative determination of anti-OVA IgGs was done by parallel line analysis comparing each serum to the standard - monoclonal anti-OVA IgG - to which we voluntarily assigned 20 000 arbitrary units per mL (AU/mL).

For quantification of OVA specific immunoglobulin G subclass, IgG1 and IgG2a, plates were coated with OVA as described above and incubated with sera and standard antibody preparations (IgG1 or IgG2a). Plates were washed and 100 μL of biotinylated rat anti-mouse IgG1 at 0.05 μgmL^{-1} or biotinylated rat anti-mouse IgG2a at 0.5 μgmL^{-1} were added to each well and incubated 2 h at 37 ºC. After washing, avidin-HRP (50 000 × diluted) was added for determination of IgG1, while streptavidin-HRP (100 000 × diluted) was used for IgG2a determination (100 μL per well) and incubated 2 h at 37 ºC. Plates were washed and supstrate solution was added as describe above. The enzymatic reaction was stopped with H_2SO_4 and absorbency at 492 nm was measured using a microplate reader.

The relative quantities of antibody subtypes were determined by parallel line assay using appropriate standard preparation. The monoclonal anti-OVA IgG1 was a standard for relative quantification of anti-OVA IgG1 to which 400 000 AU/mL was assigned, while polyclonal mouse serum containing high levels of anti-OVA IgG2a was used as a standard for relative quantification of IgG2a specific antibodies with voluntarily assigned 5 000 AU/mL.

3. Acellular ROS activity testing

The ability to generate extracellular reactive oxygen species was examined by spectrophotometric method with DCFH2-DA. Solutions of certain analyte concentrations (250 mgL^{-1}, 125 mgL^{-1}, 62.5 mgL^{-1}, 31.3 mgL^{-1}, 15.6 mgL^{-1}, 7.8 mgL^{-1}, 3.9 mgL^{-1}) were prepared in DMSO. The method consists of measuring the change in fluorescence intensity of the compound DCFH2-DA, 2,7-dichlorodihydrofluorescein, which does not fluoresce, but in the presence of ROS species is oxidized to DCF, 2,7-dichlorofluorescein and fluoresces. 1300 μl of 1 mM DCFH2-DA solution (prepared from 10 mM "stock" solution) was hydrolyzed with 5.2 ml 0.01 M NaOH for 30 min at room temperature in the dark. Hydrolysis was stopped with 19.5 ml of 0.1 M PBS, and the resulting 50 μM solution was diluted with 0.01 M PBS to a concentration of 10 μM. The thus prepared fluorescent dye was added to the prepared analyte concentrations on a microtiter plate (96 "well" plate), and the measurement was performed on a Victor microtiter plate reader Spectrometer, Perkin Elmer at wavelengths of 485 nm for excitation and 530 nm for emission, for 90 min. SIN-1, 3-morpholine sydnonimine hydrochloride was used as positive control. Interference tests were also performed. The autofluorescence test was performed by measuring the change in fluorescence intensity in PBS, and the fluorescence quenching test was performed with a 0.1 μM F-DA (fluorescein diacetate) solution.

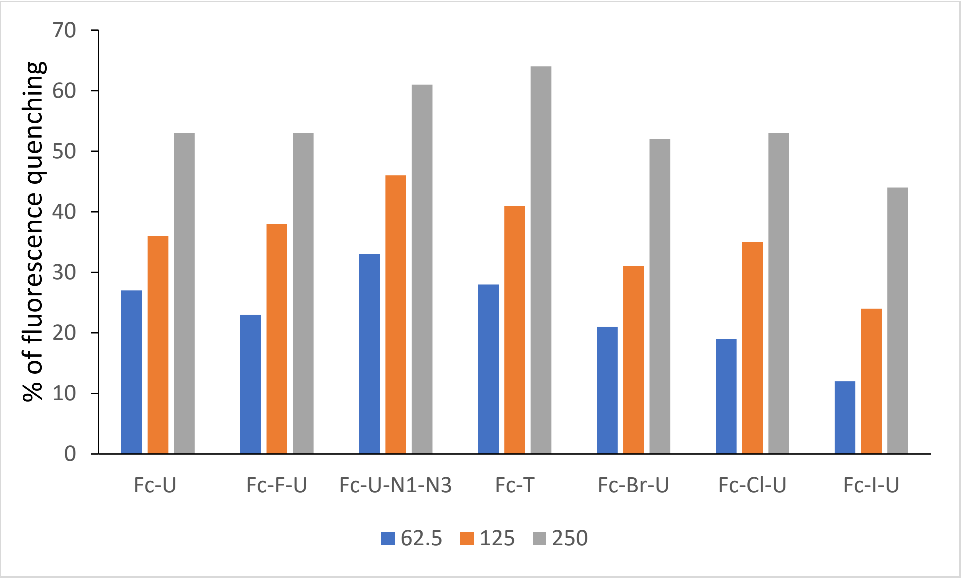

Figure S1. Fluorescence quenching test using a 0.1 μM F-DA (fluorescein-diacetate solution). Results are shown for the tested compounds at concentrations of 62.5, 125, and 250 mgL^{-1}.

The fluorescence quenching assay (Figure S1) revealed that ferrocenoylated uracil derivatives exhibit varying capacities to quench fluorescence, indicative of their interaction strength with the fluorophore and potential redox activity. Although the differences in fluorescence quenching among the compounds are relatively modest, the observed activity confirms the electron-donating or -accepting properties of tested compounds. These findings imply a structure-dependent redox behavior, where enhanced fluorescence quenching aligns with greater ROS-generating potential. Such properties highlight these compounds as promising candidate for further exploration in redox-based therapeutic applications, including anticancer strategie.

4. IR and NMR Spectra

Figure S2. a) ^{1}H NMR and b) ^{13}C APT NMR of compd. **4d**

a)

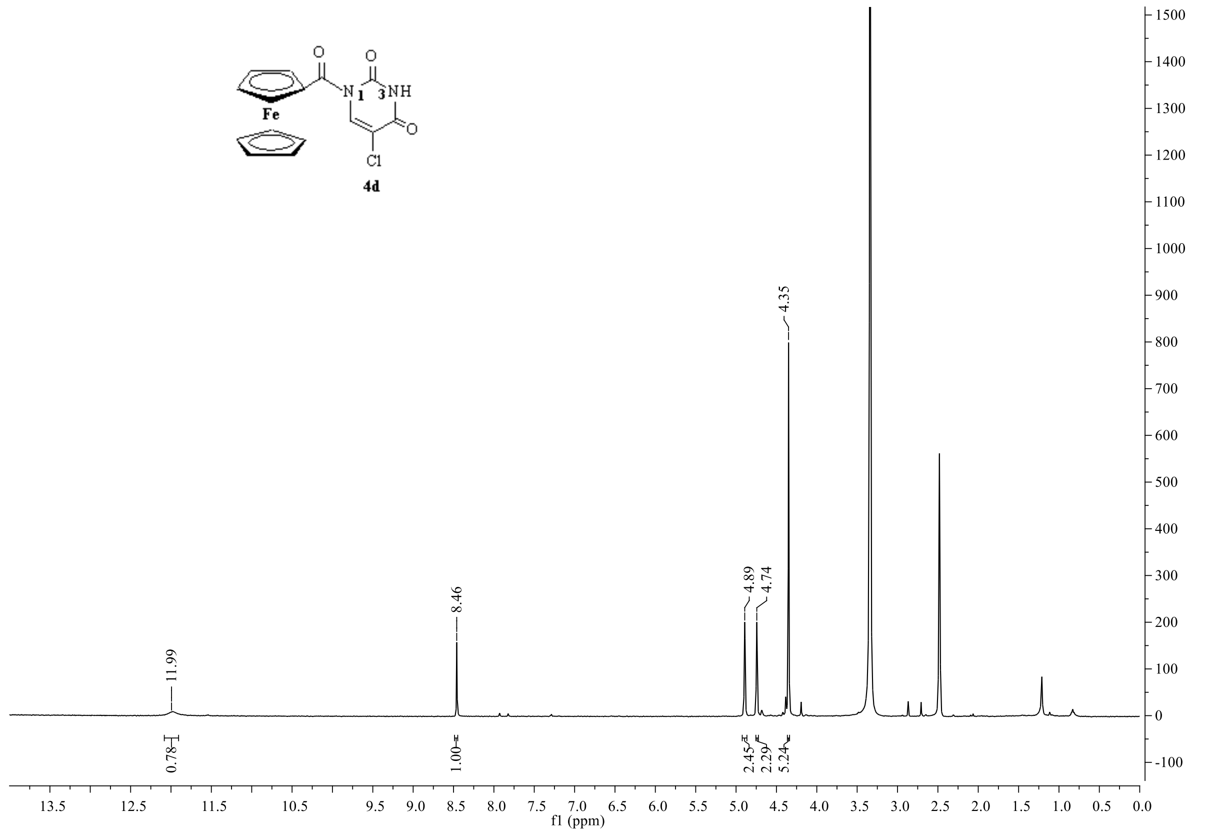

b)

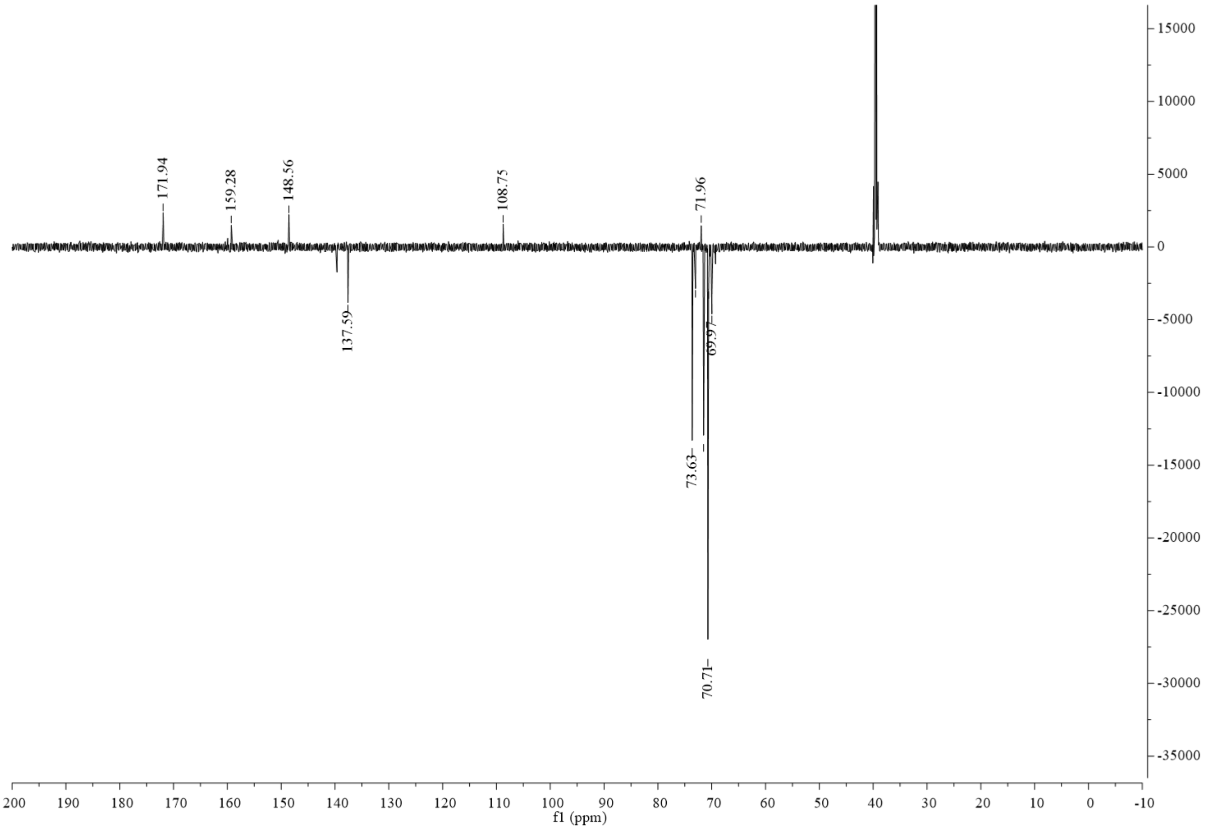

Figure S3. a) ^{1}H NMR and b) ^{13}C NMR of compd. **4e**

a)

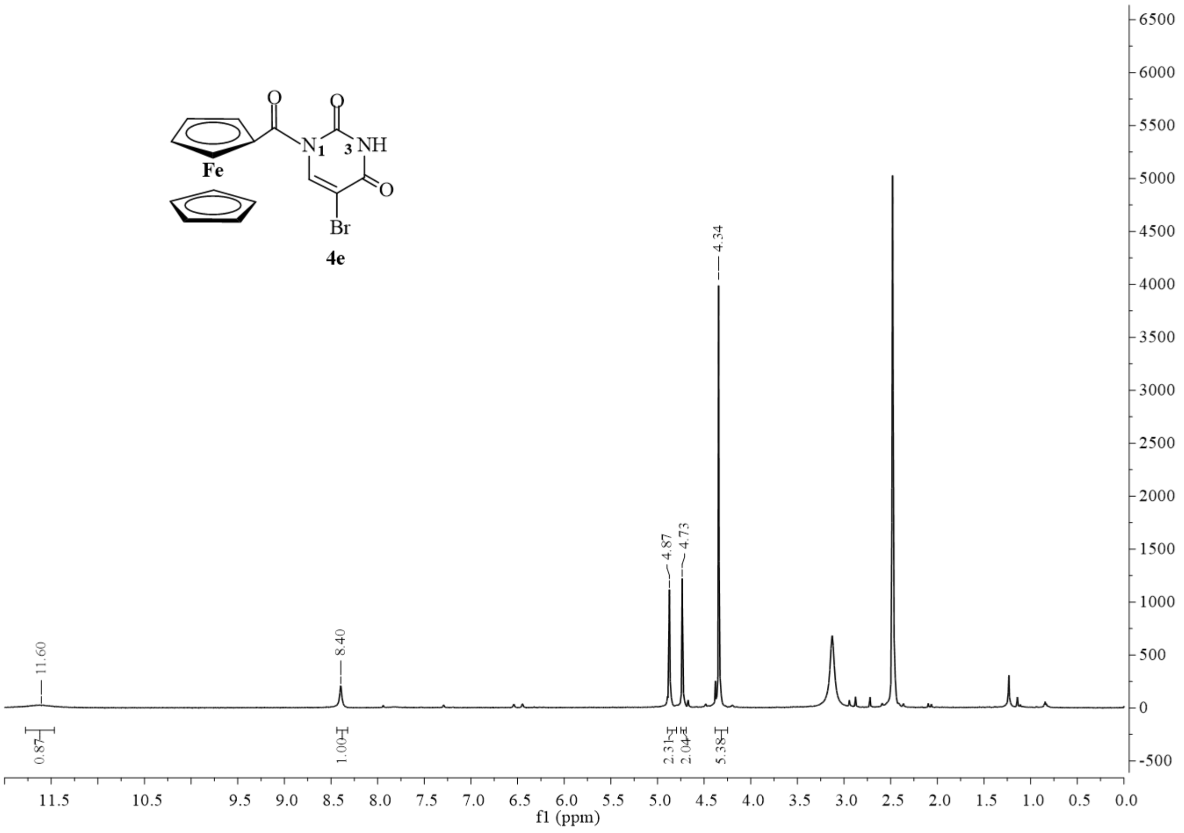

b)

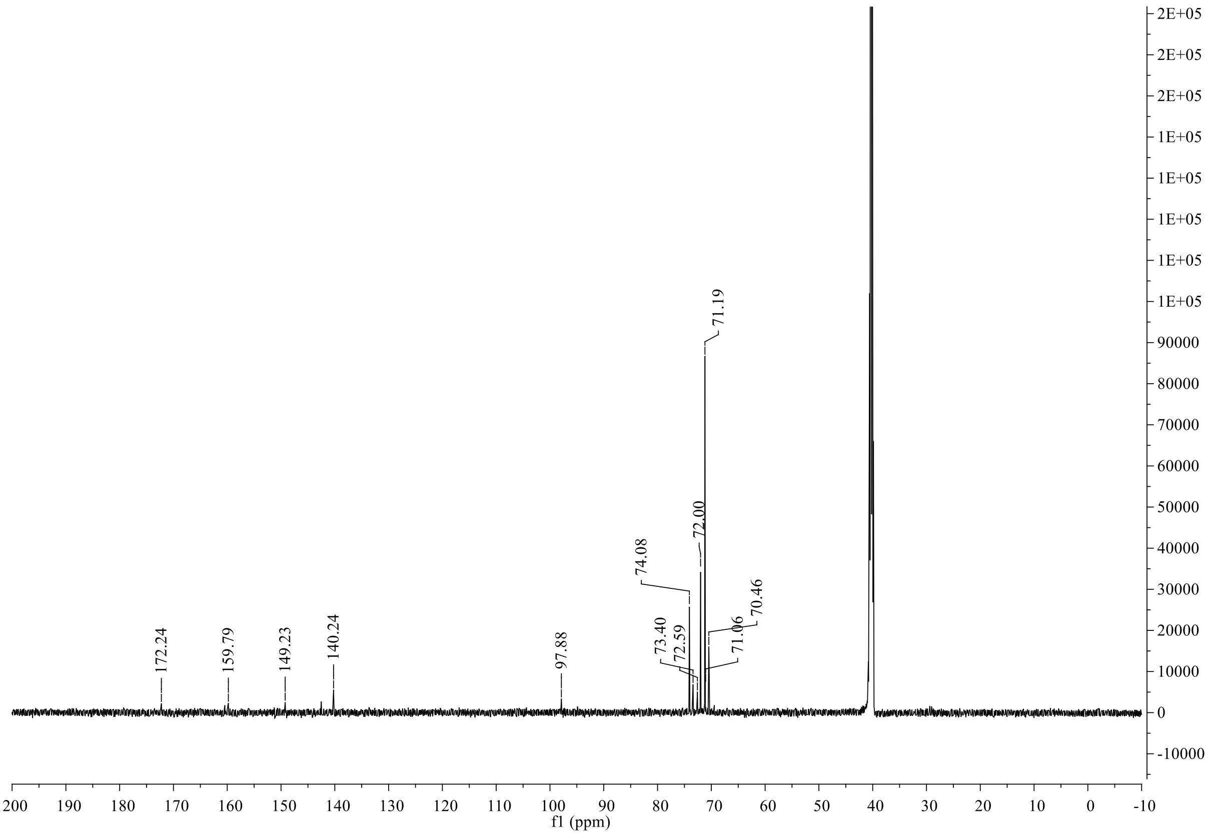

Figure S4. a) ^{1}H NMR and b) ^{13}C APT NMR of compd. **4f**

a)

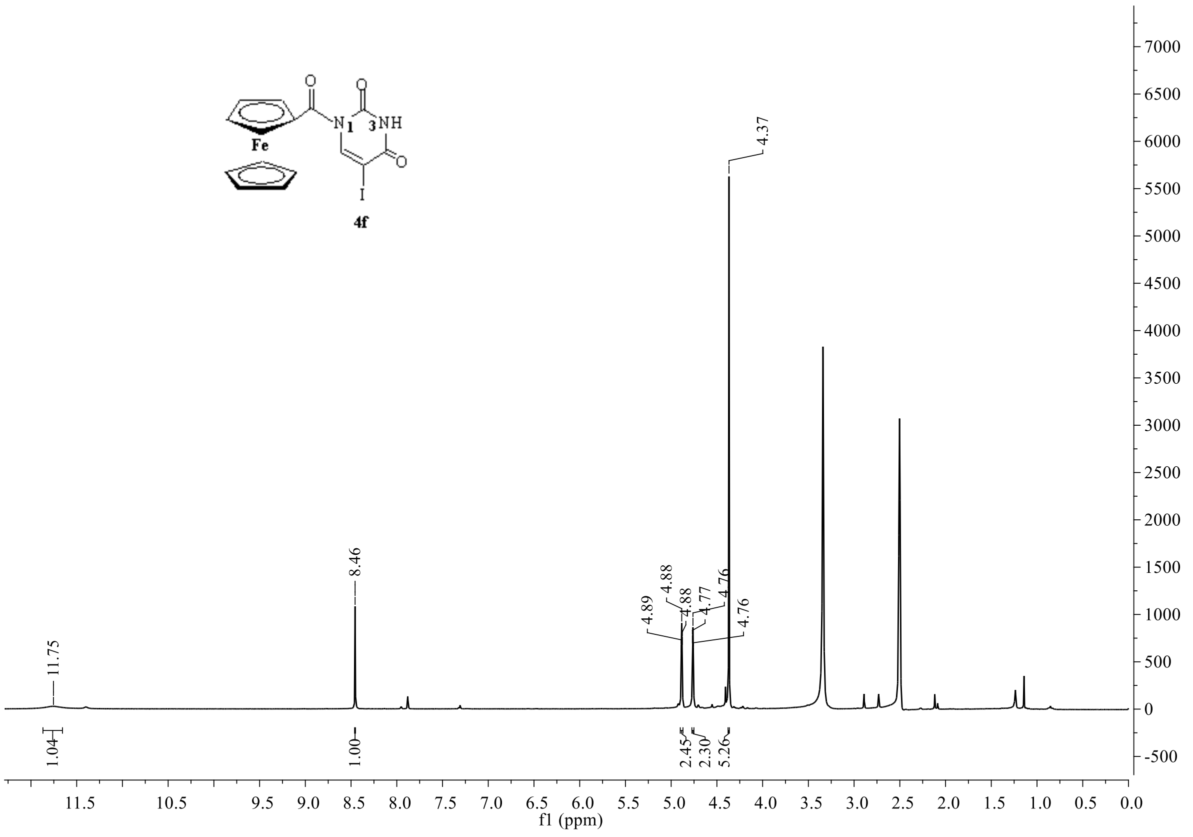

b)

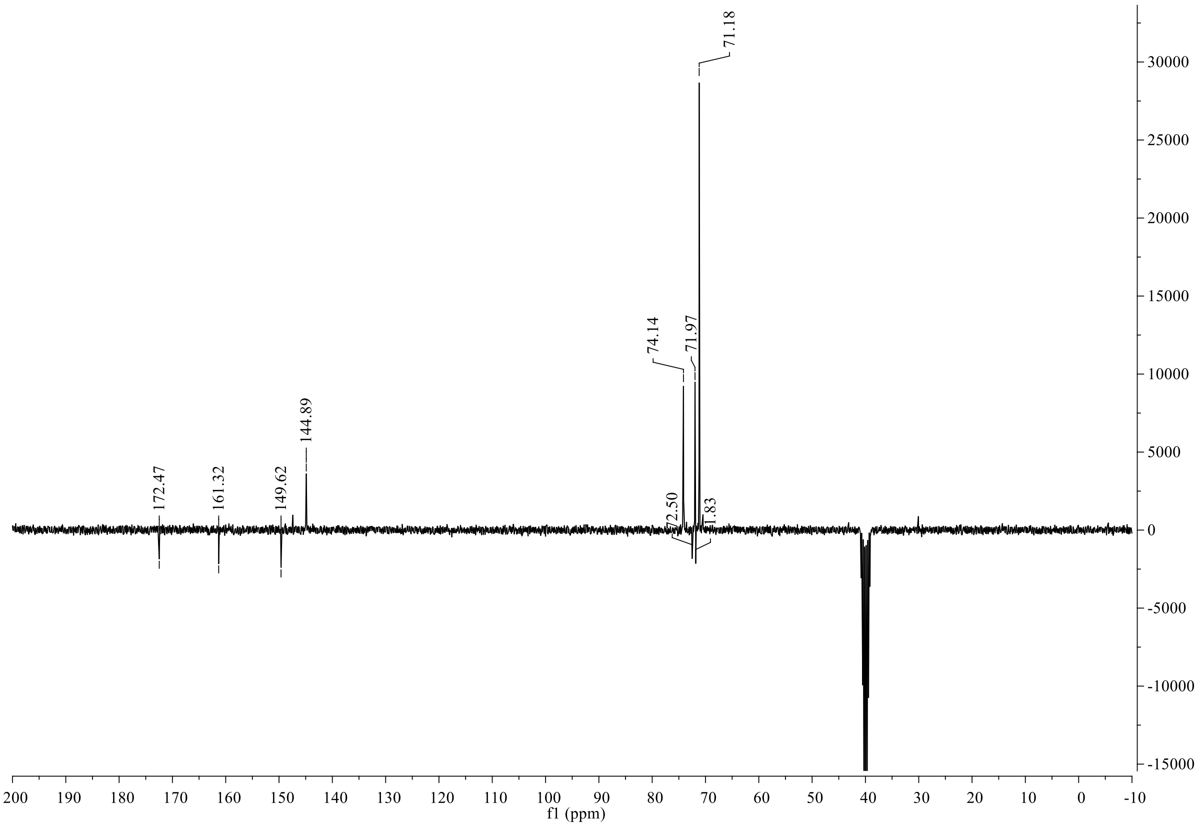

Figure S5. a) ^{1}H NMR and b) ^{13}C NMR of compd. **6a**

a)

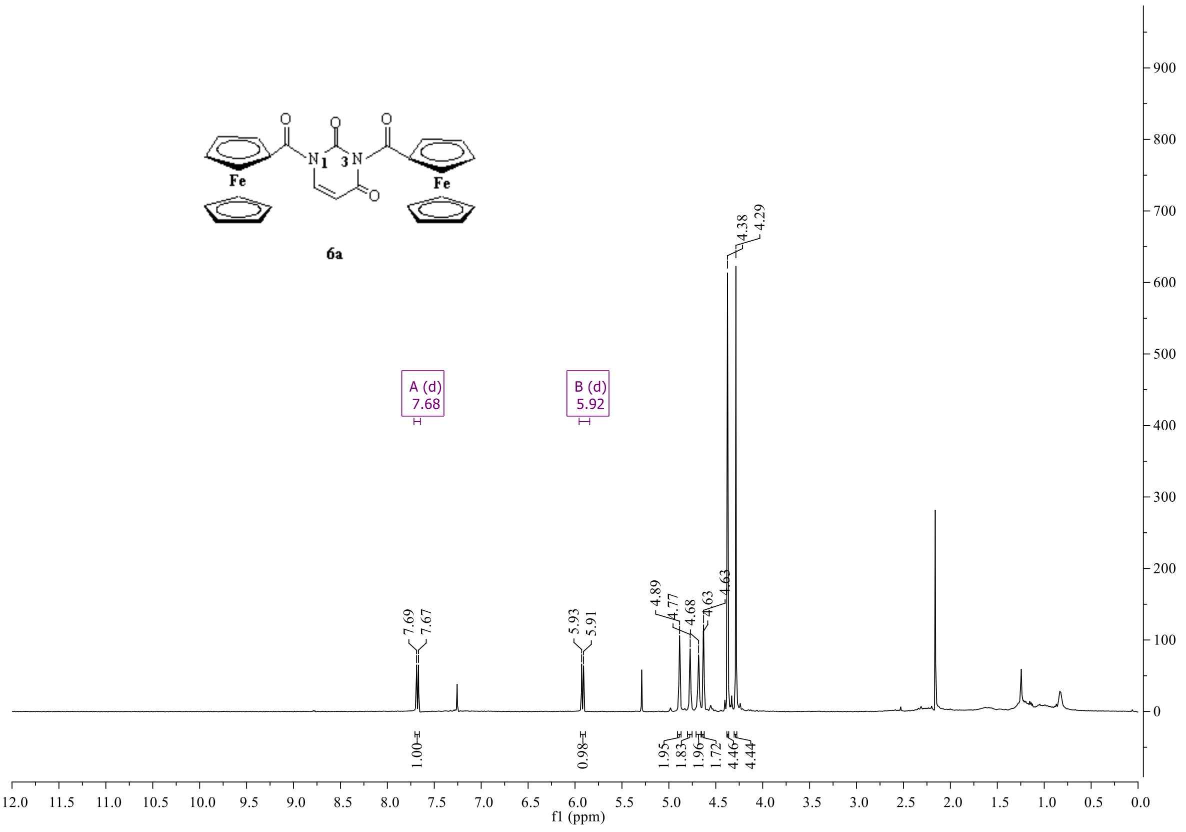

b)

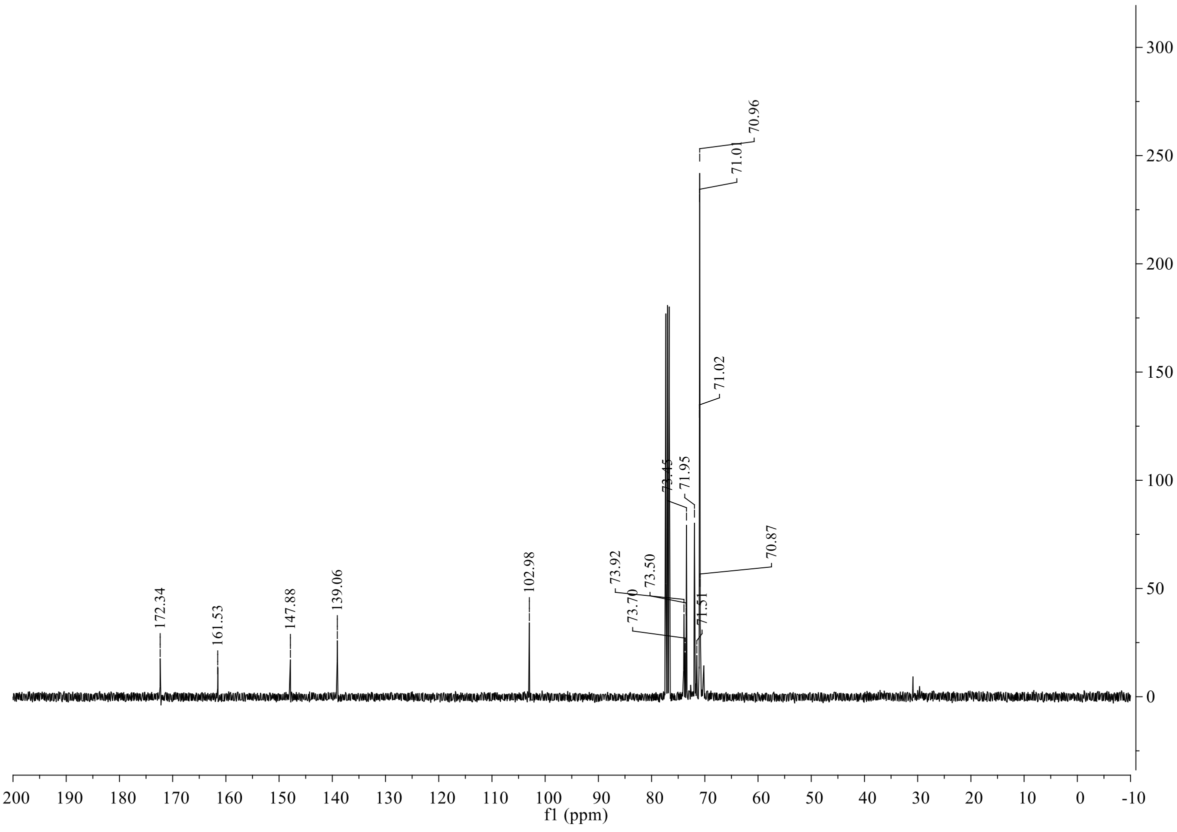

Figure S6. a) ^{1}H NMR and b) ^{13}C NMR of compd. **6b**

a)

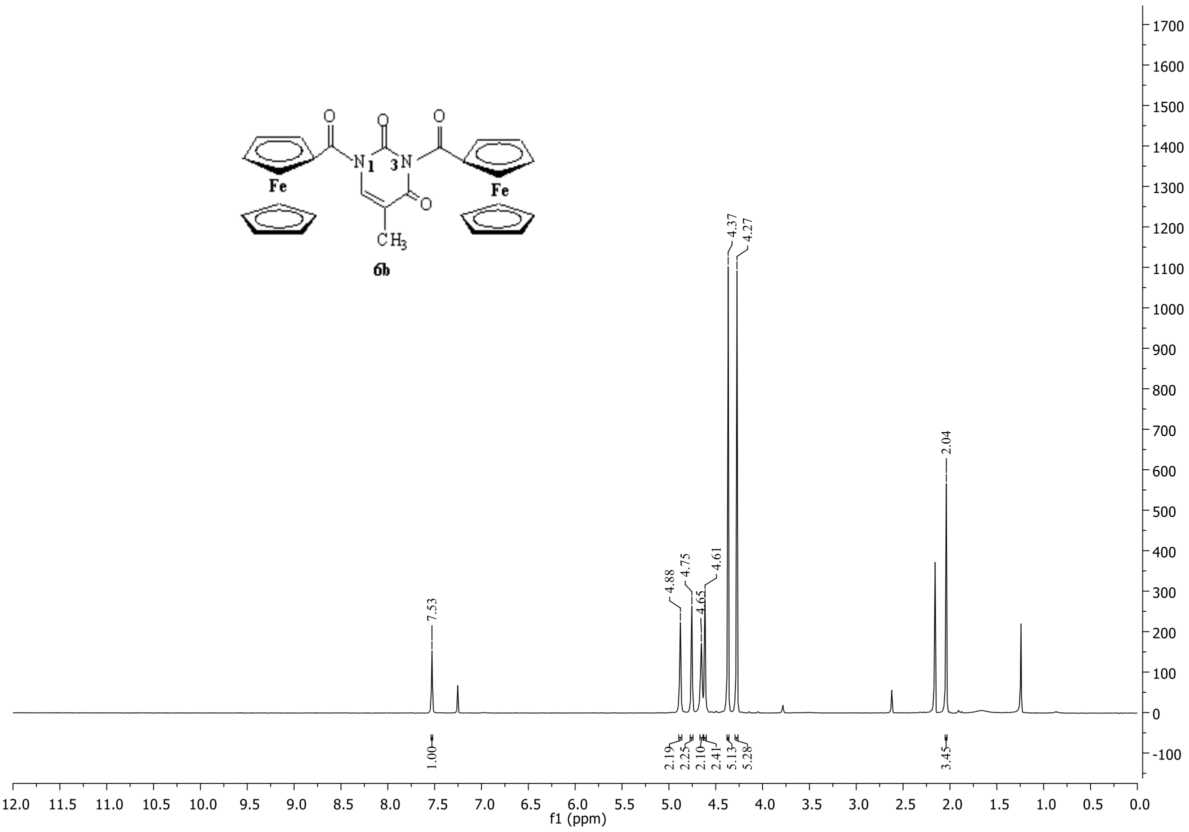

b)

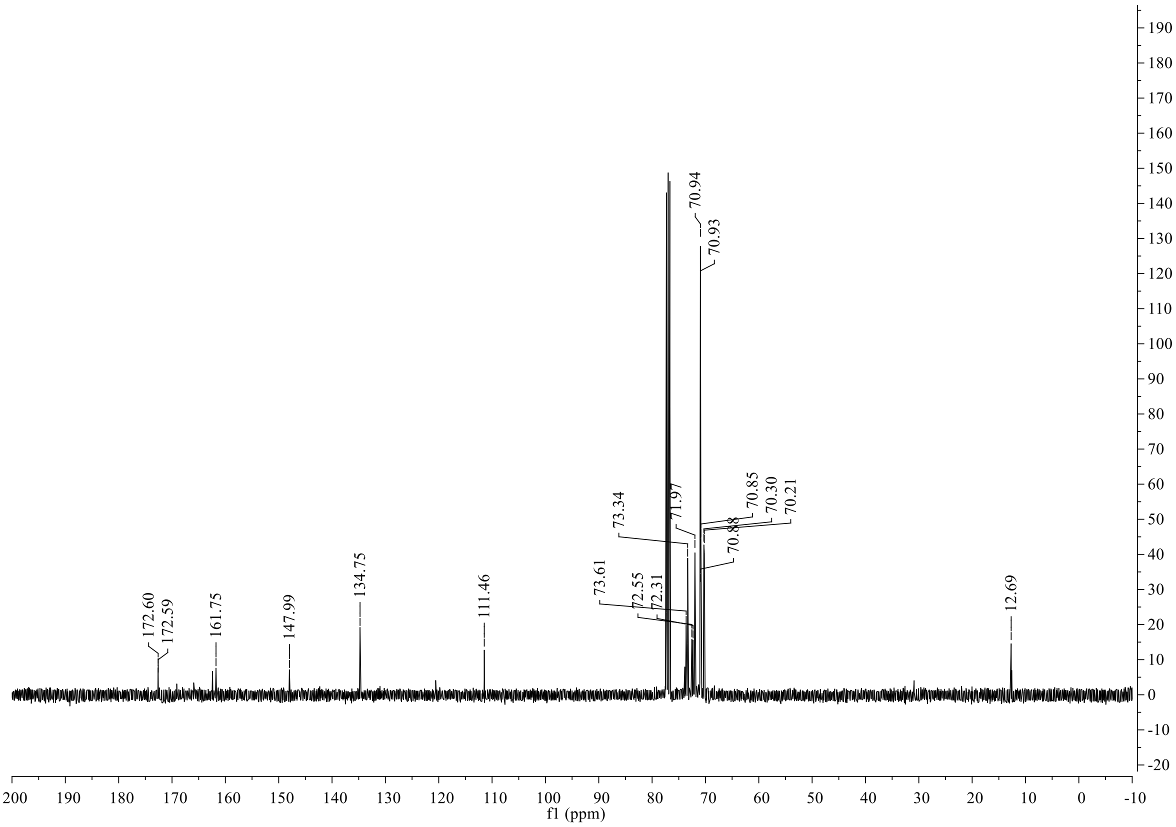

Figure S7. a) ^{1}H NMR and b) ^{13}C APT NMR of compd. **6c**

a)

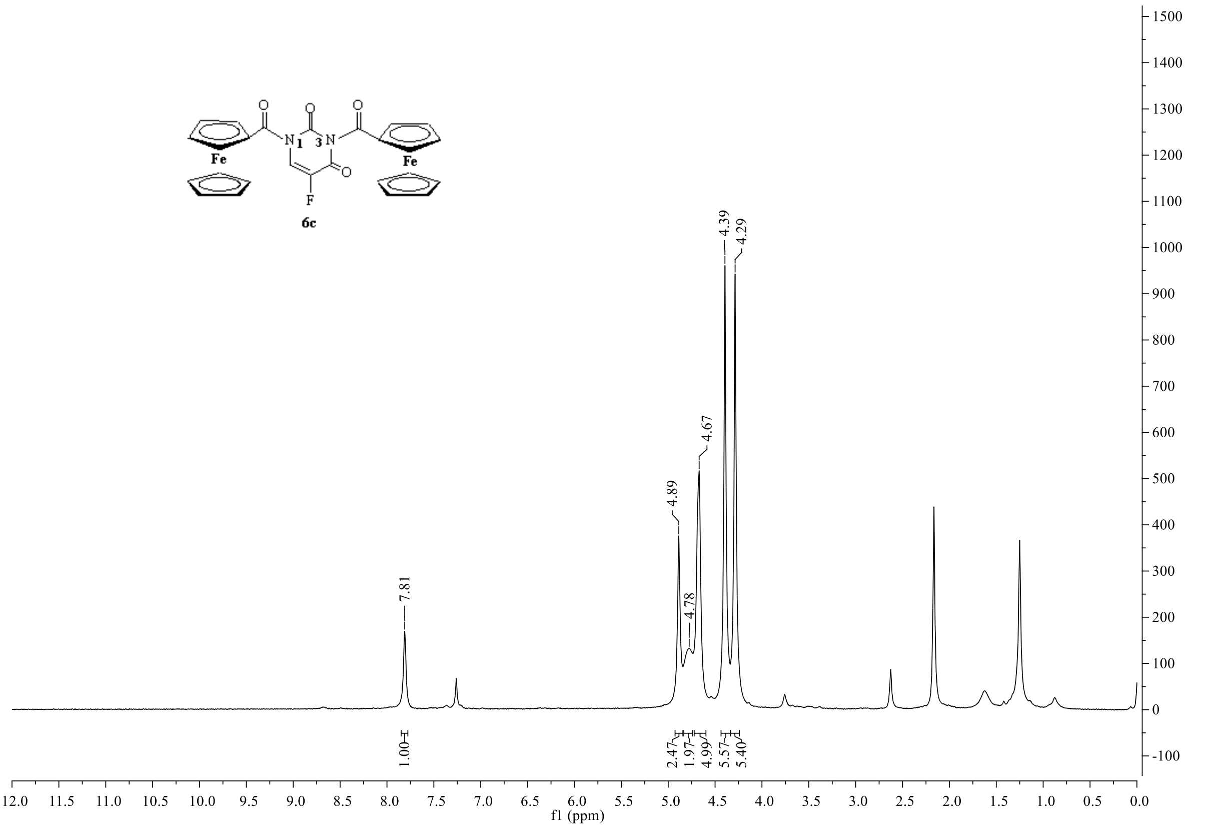

b)

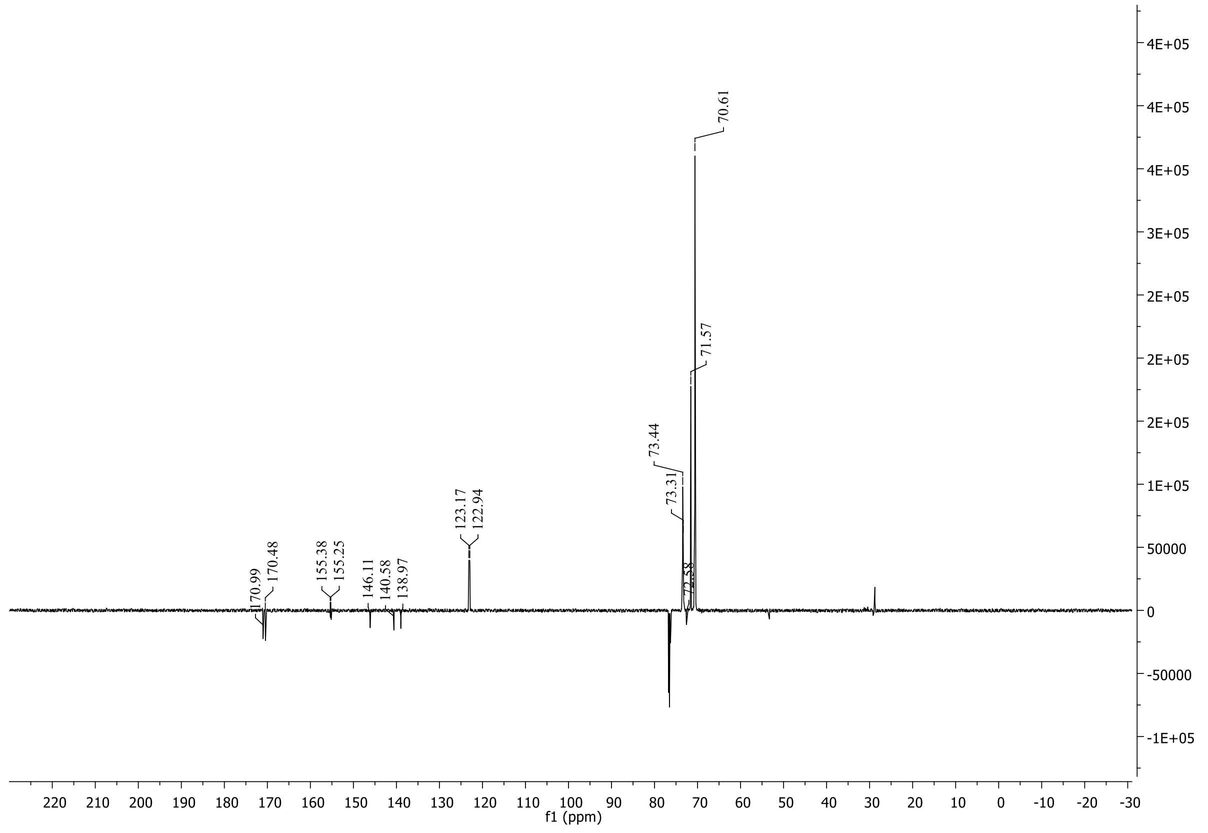

Figure S8. a) ^{1}H NMR and b) ^{13}C NMR of compd. **6d**

a)

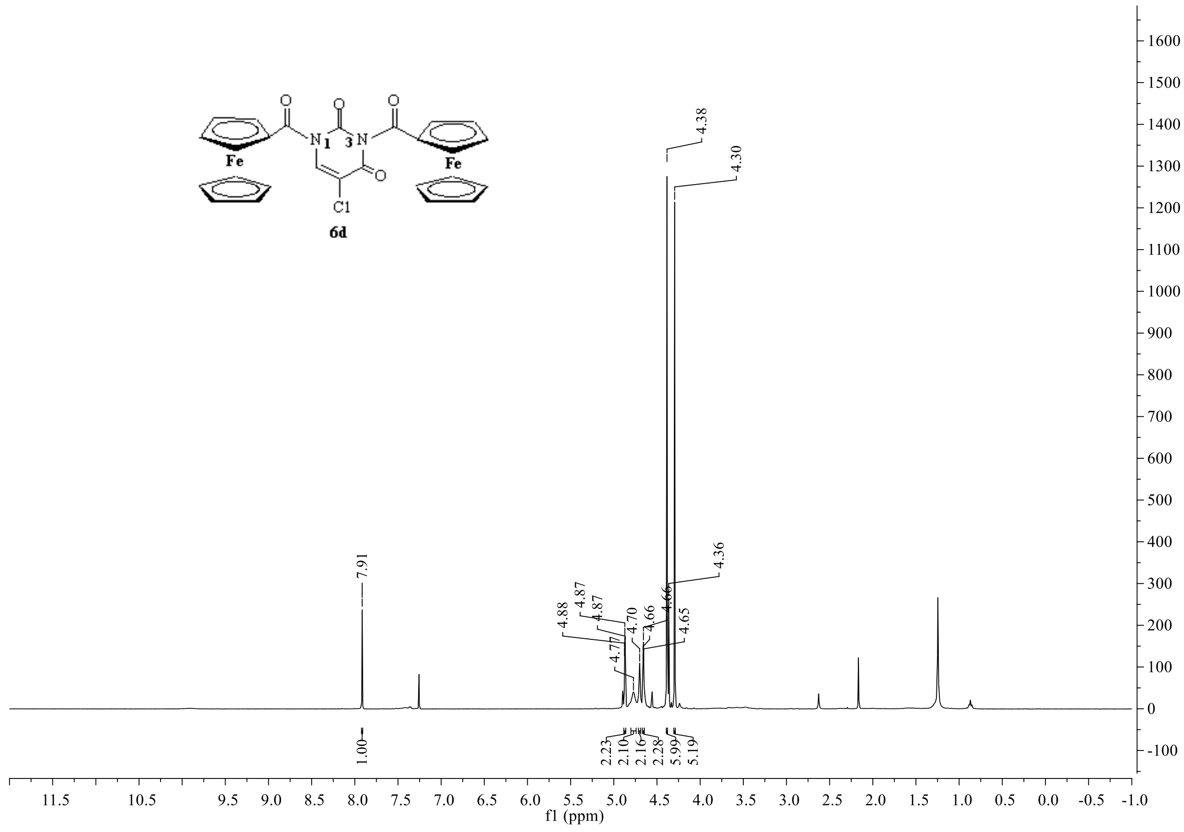

b)

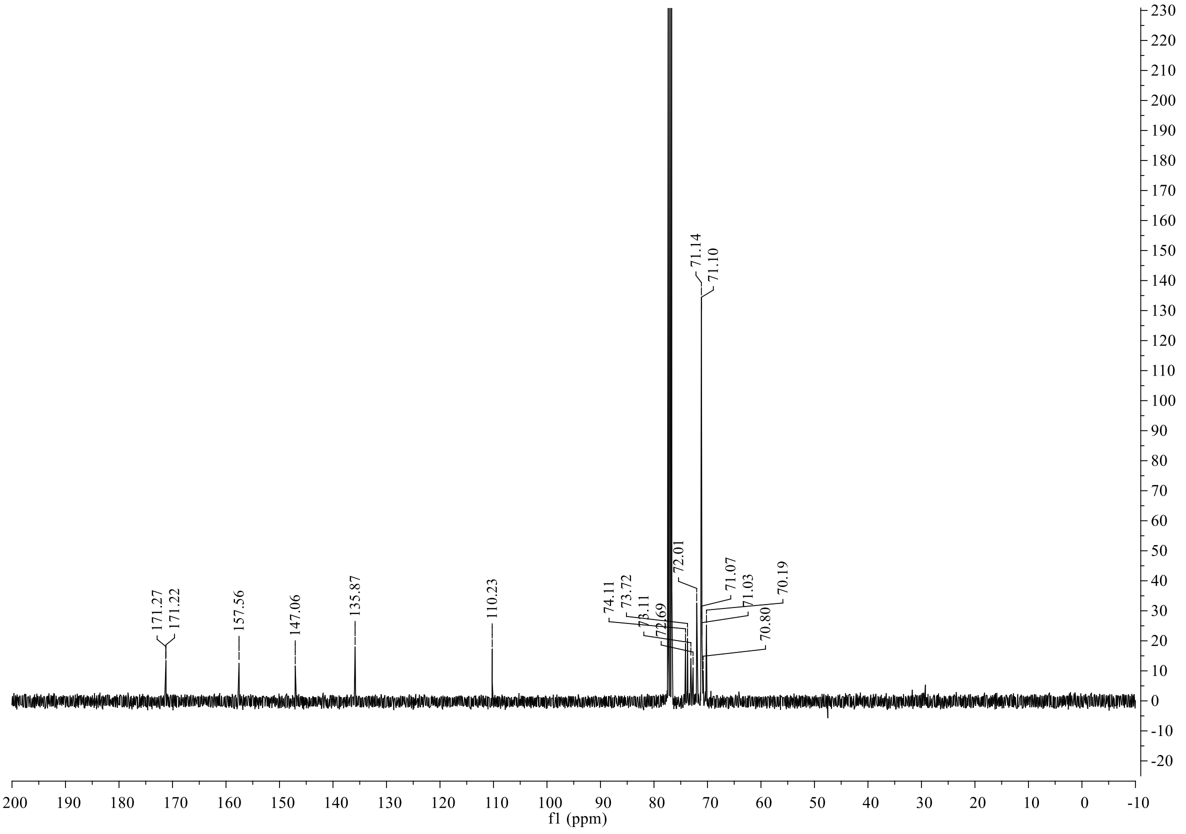

Figure S9. a) ^{1}H NMR and b) ^{13}C NMR of compd. **6e**

a)

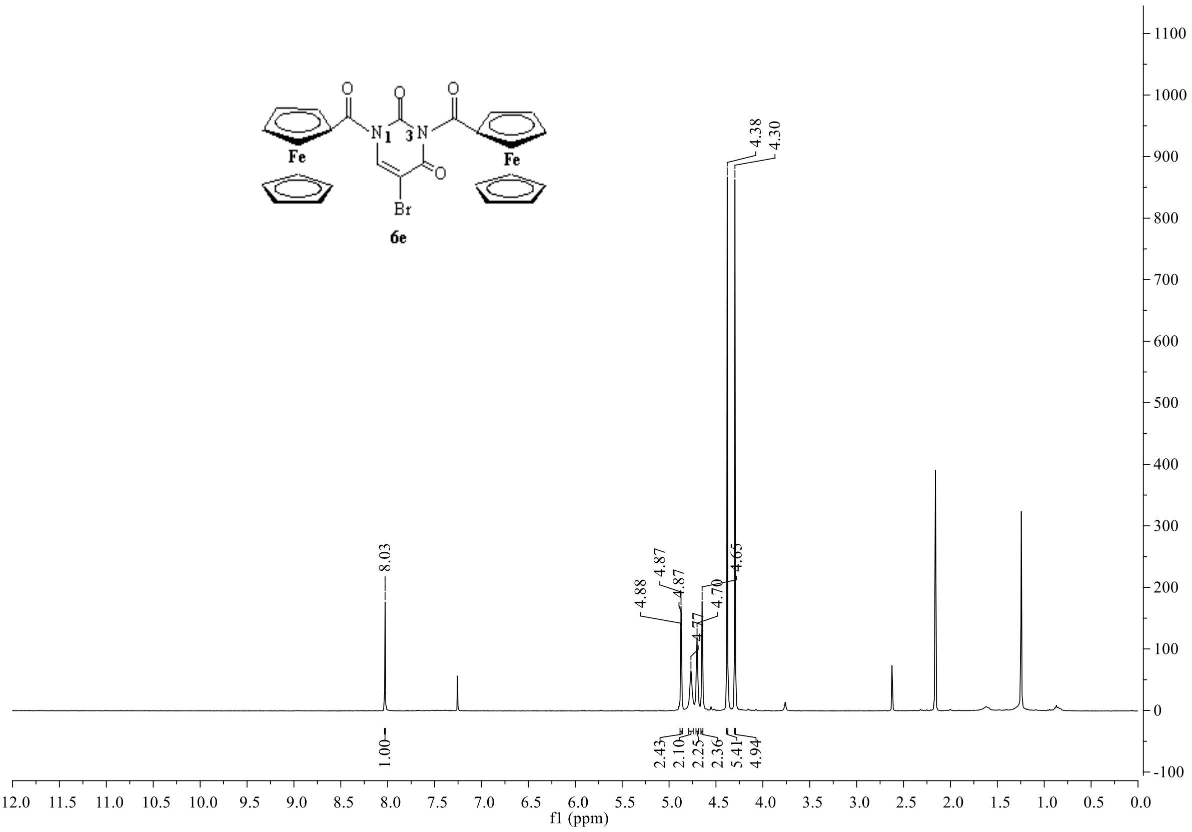

b)

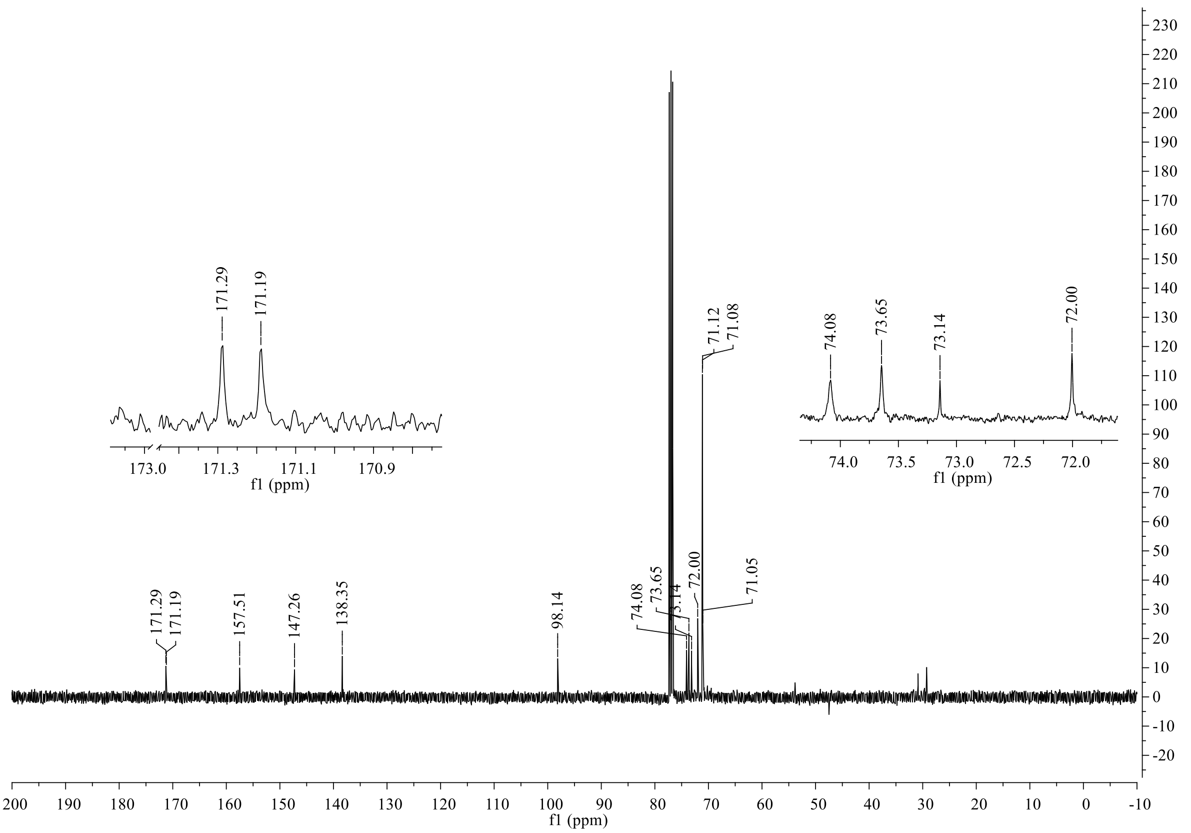

Figure S10. a) ^{1}H NMR and b) ^{13}C APT NMR of compd. **6f**

a)

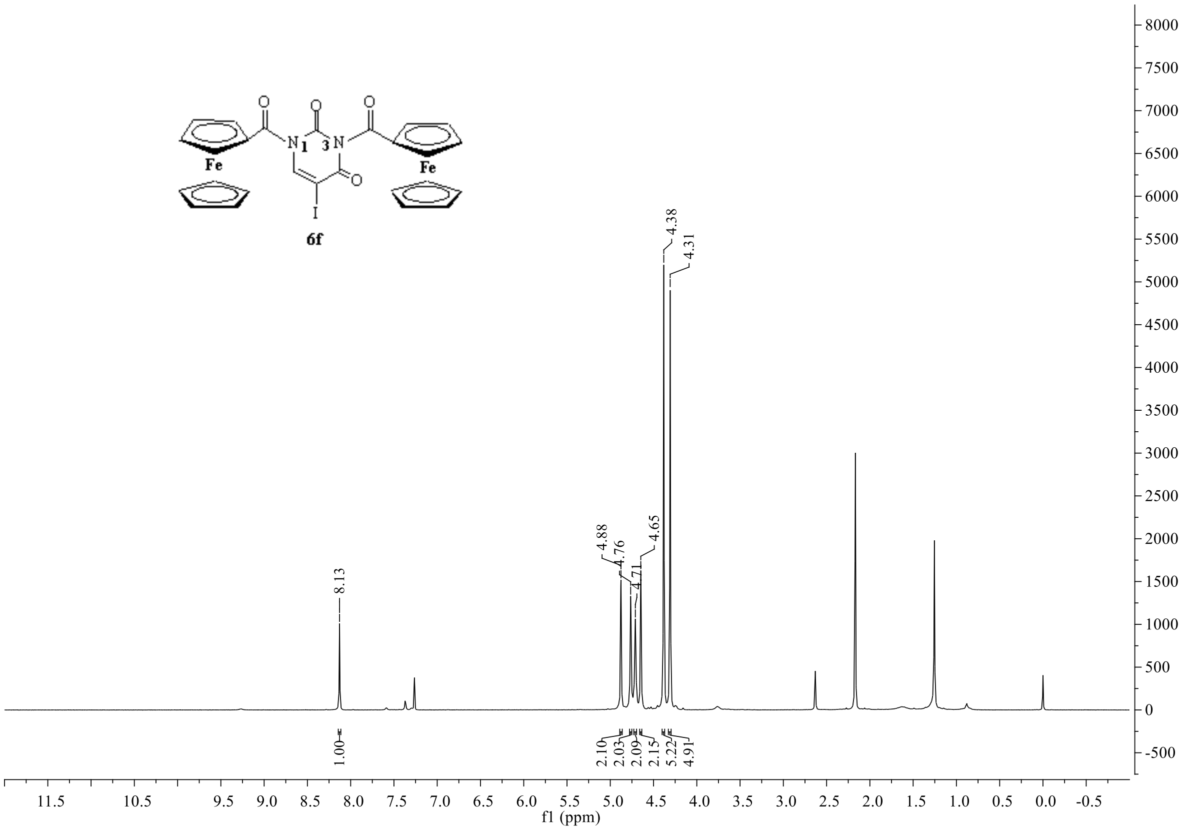

b)

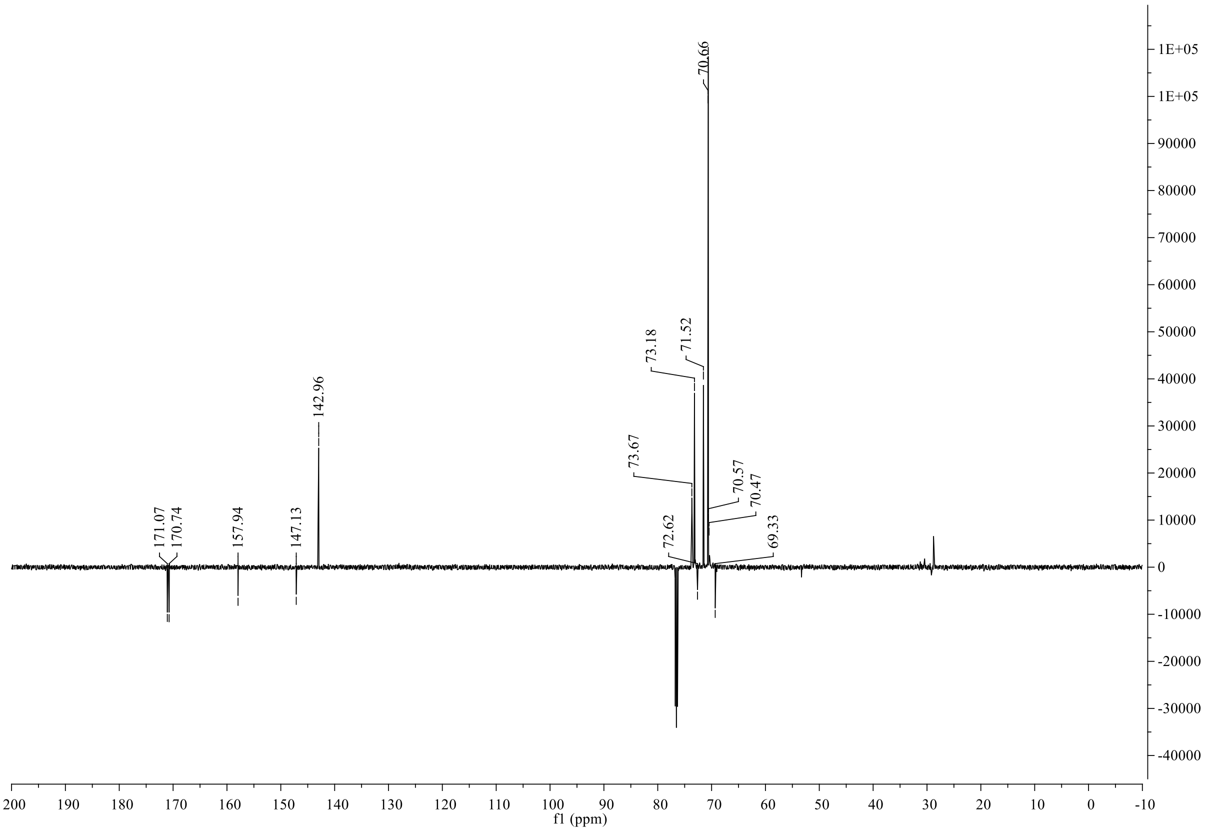

Figure S11. IR spectra of compd. **4a** i **6a**

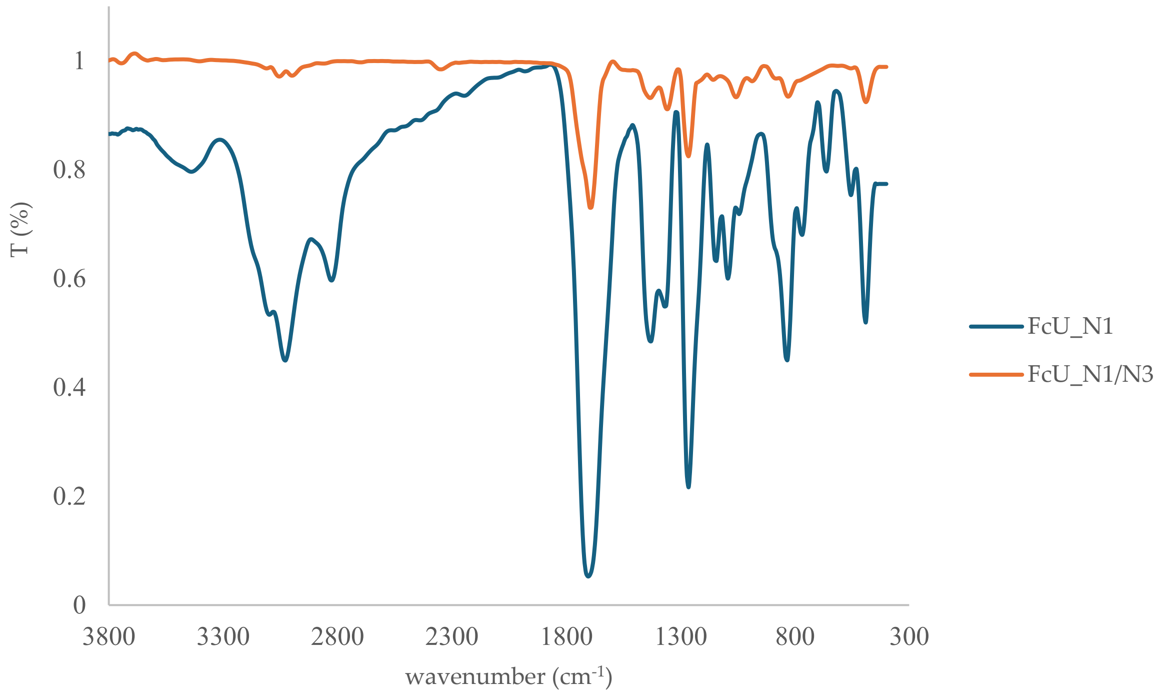

Figure S12. IR spectra of compd. **4b** i **6b**

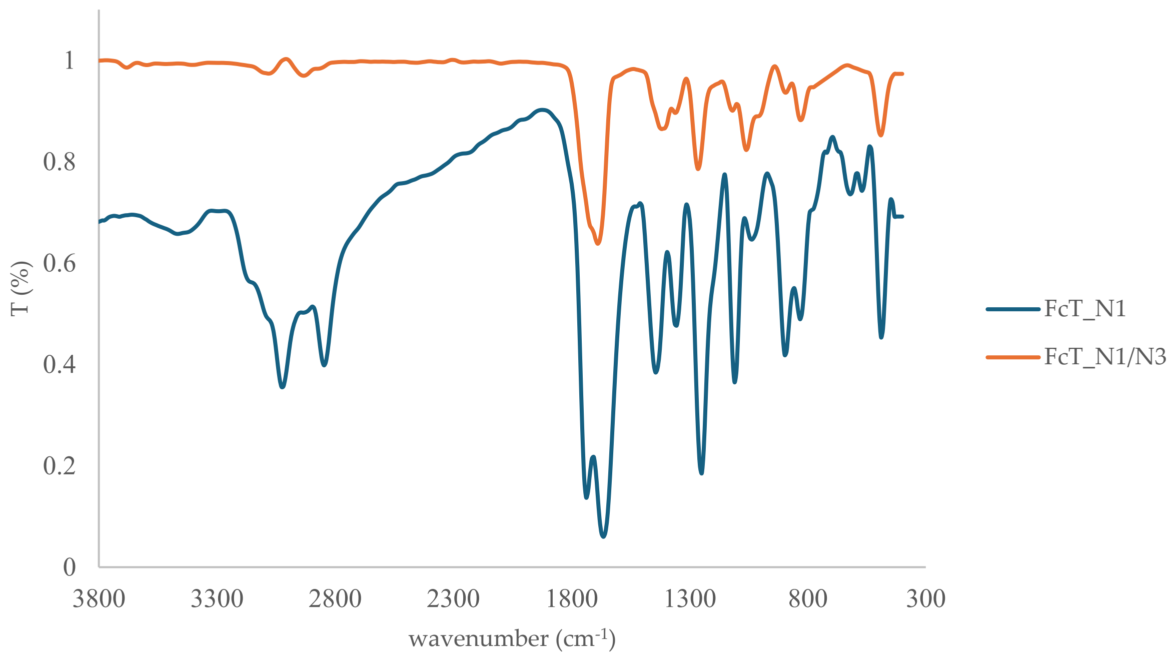

Figure S13. IR spectra of compd. **4c** i **6c**

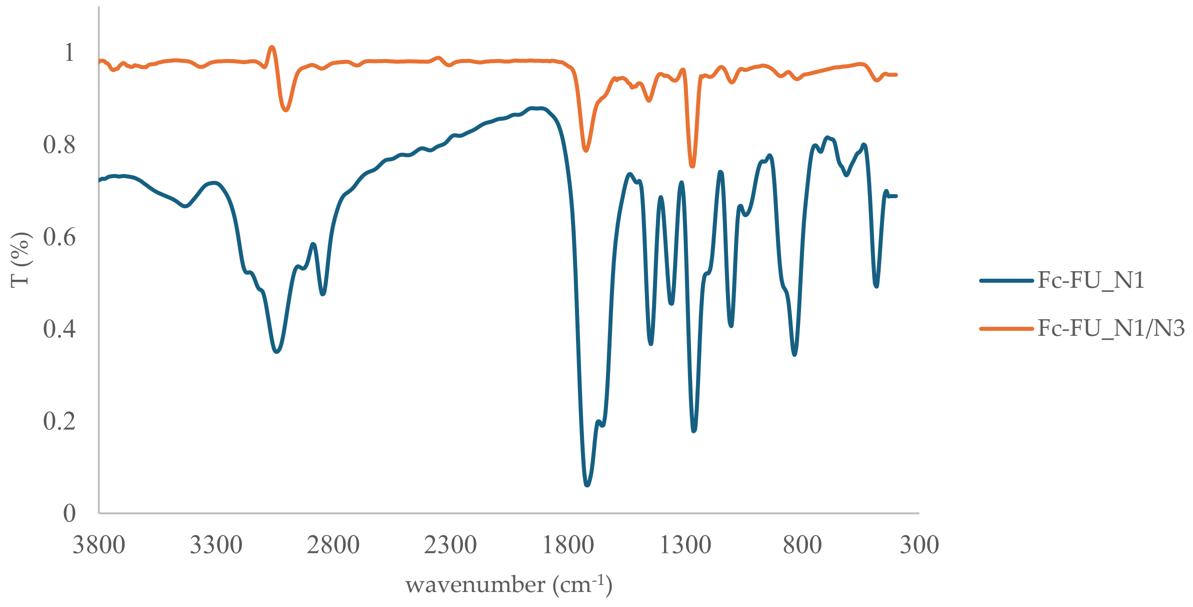

Figure S14. IR spectra of compd. **4d** i **6d**

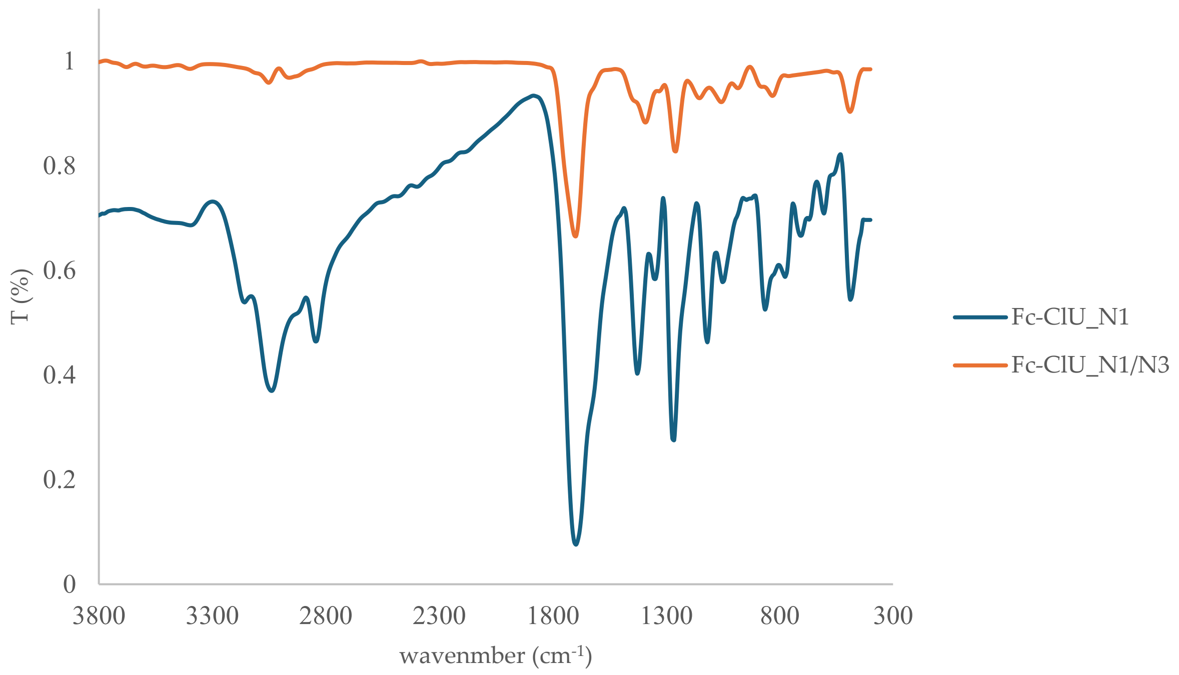

Figure S15. IR spectra of compd. **4e** i **6e**

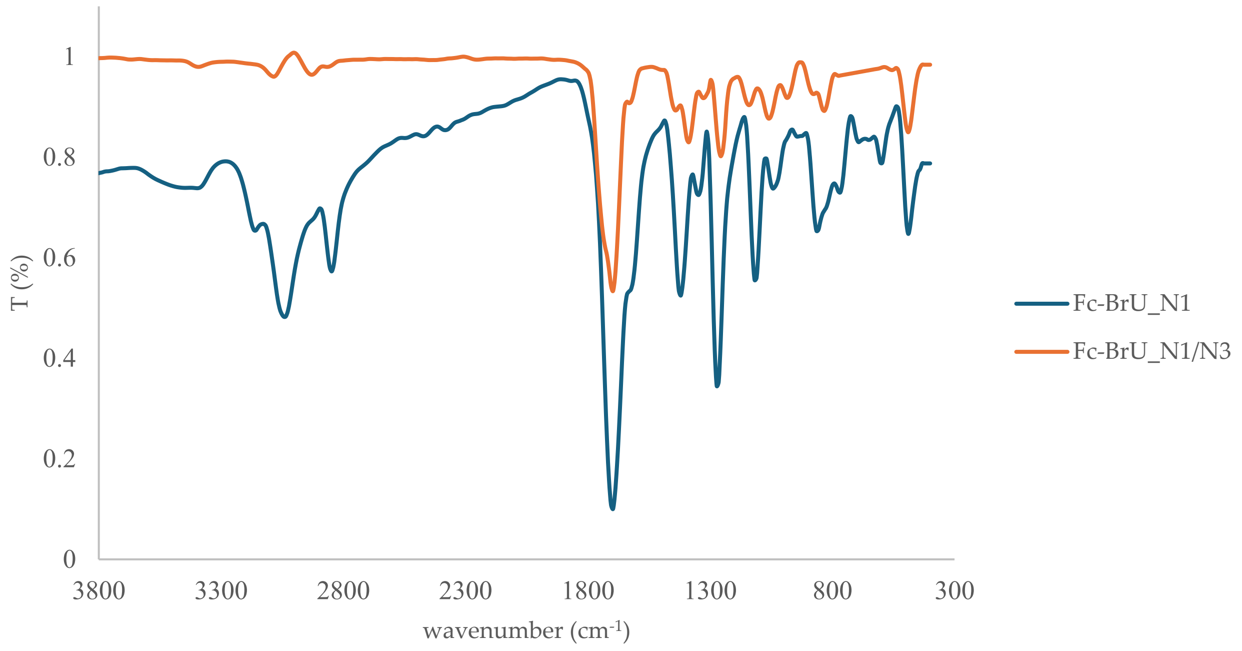

Figure S16. IR spectra of compd. **4f** i **6f**

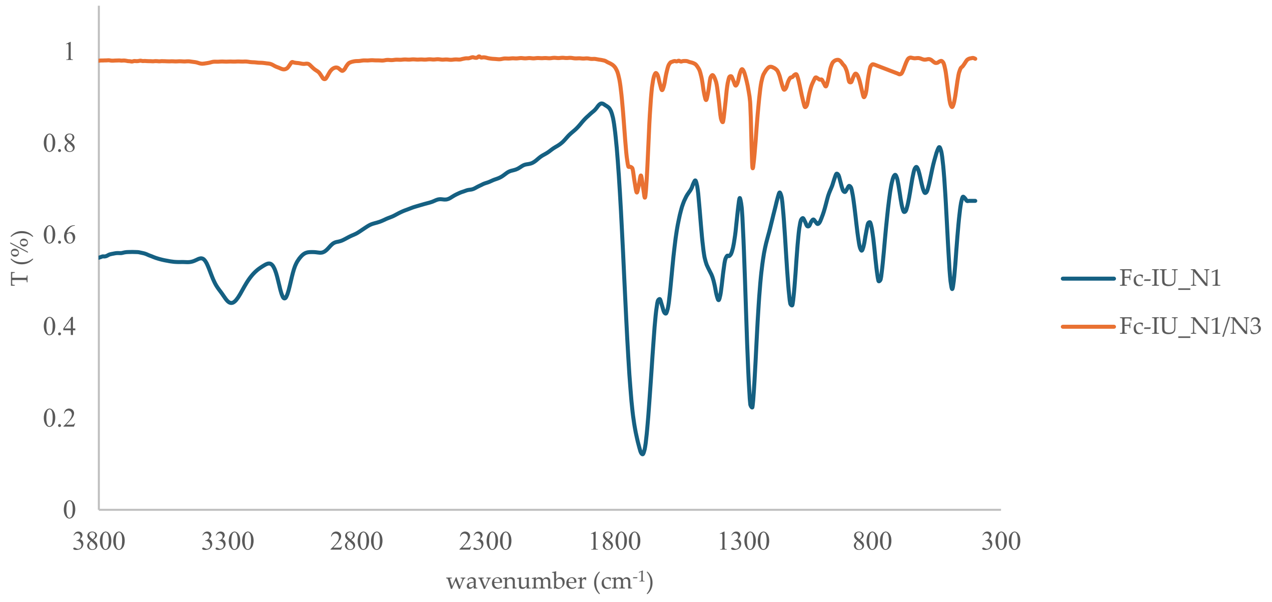

5. XYZ Coordinates of optimized stationary points

(Gibbs free energies in parentheses, in Hartrees)

DMF

(-248.438305)

C,0,3.2289571589,0.0025290373,0.5163849724

N,0,4.4279583197,-0.1247584507,-0.1257251871

O,0,2.3001838213,-0.7857819284,0.4533036569

H,0,3.1835902145,0.9286579747,1.1229375799

C,0,5.4775321964,0.8647758941,0.0078366608

H,0,5.7339696203,1.3030944036,-0.9661683639

H,0,5.1430483546,1.6680642565,0.6707197409

H,0,6.3879581779,0.4208534291,0.4328755011

C,0,4.6813559636,-1.2771548214,-0.9730635621

H,0,5.5447406747,-1.847042978,-0.6054642745

H,0,3.792866497,-1.9092109133,-0.9550165764

H,0,4.8873010011,-0.9628919035,-2.004658148

Uracil N1-anion

(-414.214329)

C,0,3.2188713689,-0.1944119669,0.673330343

C,0,2.1639604315,-1.6204241051,-0.8015378562

C,0,3.2158117487,-2.5137951573,-0.9203033596

H,0,1.2518835482,-1.8296176236,-1.3733904314

C,0,4.4177769977,-2.2592404936,-0.1807271995

H,0,3.1604694898,-3.3945693272,-1.5510439811

N,0,2.1041906905,-0.503106351,-0.06239239

N,0,4.3164143088,-1.0841142363,0.5787504811

O,0,3.3336364294,0.7964353411,1.4073597849

H,0,5.1293404682,-0.8372607395,1.1298622529

O,0,5.4620125184,-2.9332323406,-0.1529836441

Uracil N3-anion

(-414.191441)

C,0,3.3305911292,-0.2234952388,0.7056000019

C,0,2.1127191623,-1.6817397134,-0.8621236541

C,0,3.1825240144,-2.50176072,-0.9279354977

H,0,1.183393538,-1.8457238549,-1.4076488672

C,0,4.3981700786,-2.1763631341,-0.1378831334

H,0,3.1837020854,-3.3961178043,-1.5423665809

N,0,2.1659704016,-0.5671826501,-0.0702474564

N,0,4.3958449077,-1.042435895,0.638982216

O,0,3.2276566864,0.8199663342,1.3727159621

O,0,5.3804466044,-2.9404113389,-0.2043369896

H,0,1.3935477919,0.0770170453,0.0100928192

1

(-1083.805571)

Fe,0,-2.0498425963,-0.2366957404,-0.0834926995

C,0,-0.1864652995,-0.9535915456,0.4163413522

C,0,-1.2024711901,-1.6824474027,1.1341984201

C,0,-2.0343343109,-2.3160312941,0.1760139369

C,0,-1.5534785649,-1.982261506,-1.1296813391

C,0,-0.4196557074,-1.1394781683,-0.9913980752

C,0,-2.3970689277,1.5886896489,-1.016671376

C,0,-3.5443990032,0.7468895192,-1.1386179202

C,0,-3.9999585635,0.4278692985,0.175945778

C,0,-3.1348045739,1.0722007221,1.1122739977

C,0,-2.1463963808,1.7899895763,0.3739372257

H,0,-1.2977723475,-1.718829933,2.2102892738

H,0,-2.9048012948,-2.9208290789,0.3944204375

H,0,-1.9983878385,-2.292176469,-2.0662070853

H,0,0.1652842994,-0.7047175972,-1.7886663556

H,0,-1.8037729051,1.9813877371,-1.8319427565

H,0,-3.9748975504,0.3879147192,-2.0645397518

H,0,-4.835782366,-0.2150921956,0.4199565972

H,0,-3.1984892659,1.005560191,2.1904747474

H,0,-1.3245251208,2.3546605765,0.7952631241

C,0,0.805332177,-0.1243247922,1.0921552331

O,0,0.9450954388,0.0209043491,2.2699792008

Cl,0,1.9382378421,0.7613454249,-0.0620590753

TS_I

(-1332.190207)

Fe,0,2.043613685,0.3898228474,0.0728289865

C,0,0.1282988258,0.9892790799,0.4787869947

C,0,1.0700377988,1.7875874509,1.2281322263

C,0,1.8970888748,2.4612741406,0.2881667133

C,0,1.4834441252,2.0792110894,-1.0271633058

C,0,0.3909730173,1.1763736135,-0.9155126885

C,0,2.4445822096,-1.4568445071,-0.7819478676

C,0,3.5645543698,-0.5861521647,-0.9493748133

C,0,4.0180393605,-0.1946600982,0.3472953449

C,0,3.1784661754,-0.8271274657,1.3159045471

C,0,2.2075223122,-1.6052421413,0.6163819919

H,0,1.1186418468,1.8495268786,2.3066468804

H,0,2.722020747,3.1190128593,0.5299002848

H,0,1.9517972785,2.3907375103,-1.9518000867

H,0,-0.1269578384,0.6597810524,-1.7101089211

H,0,1.8336548633,-1.8853943642,-1.5645394501

H,0,3.9769935659,-0.2514586698,-1.8927941275

H,0,4.8340486494,0.4851710022,0.5576326789

H,0,3.2451059607,-0.708108457,2.3897368113

H,0,1.3826305772,-2.1536562997,1.0507967768

C,0,-0.9141092455,0.2126655853,1.138617524

C,0,-3.2953346946,-0.0196809969,0.6399298212

N,0,-4.4675737344,0.1242344742,0.0472305861

Cl,0,-1.2795789557,-1.6991915682,-0.456175618

O,0,-1.0156970901,-0.2309421364,2.2335348351

O,0,-2.3693936769,0.8456963642,0.5377106109

H,0,-3.1529931796,-0.8918573214,1.27367739

C,0,-5.4910524696,-0.910505649,0.1640453536

H,0,-5.6757290554,-1.3621755325,-0.8166356796

H,0,-5.1533074841,-1.6886333169,0.8504950571

H,0,-6.4233076606,-0.4756943388,0.5400838949

C,0,-4.7355642349,1.2325357295,-0.8673543552

H,0,-5.6219547292,1.7817047382,-0.5326737773

H,0,-3.8737677252,1.8976650003,-0.8840693365

H,0,-4.9157826491,0.8402895016,-1.874161603

I

(-871.794748)

Fe,0,-2.7973338798,-0.6007708513,0.0147819034

C,0,-0.7446208103,-0.6902425318,-0.1574037396

C,0,-1.254799413,-1.9333359342,0.3827956537

C,0,-2.1097207829,-2.495556753,-0.5933005396

C,0,-2.1655386756,-1.6139970917,-1.7191435761

C,0,-1.344783994,-0.4906581973,-1.4599235976

C,0,-3.9823680446,1.1134824081,-0.0316752318

C,0,-4.7713646204,-0.0414899377,-0.3176237794

C,0,-4.6928664431,-0.9192217812,0.8035538894

C,0,-3.8551985061,-0.3108210701,1.7864290267

C,0,-3.4212940622,0.9461923964,1.2700782918

H,0,-0.9998058406,-2.3482178087,1.3480012447

H,0,-2.6588898766,-3.4218520879,-0.4903011101

H,0,-5.3122348545,-0.2313906898,-1.2355061151

H,0,-5.1624751567,-1.8910104408,0.8823132415

H,0,-3.5854106461,-0.7337282998,2.7450958504

H,0,-2.7616360464,1.6412884377,1.7743326249

C,0,0.1110655951,0.1786025902,0.5825919141

O,0,0.6076762963,0.1243864006,1.6663822168

O,0,0.4456144336,1.4076714255,-0.282019332

C,0,1.288037258,2.2334249313,0.2517922105

H,0,1.662832377,2.0095147095,1.2498469465

N,0,1.6933574819,3.3055558523,-0.3670991101

C,0,2.6548515635,4.2203146779,0.2721601591

H,0,3.5540558268,4.2847804848,-0.3464545743

H,0,2.2029147684,5.212058242,0.3595132684

H,0,2.9199978963,3.8514102045,1.263914501

C,0,1.2352862397,3.6682336897,-1.718313094

H,0,0.5501644609,2.91035823,-2.0920865395

H,0,0.7327786351,4.6385335275,-1.6736253468

H,0,2.1049287299,3.7424475046,-2.3770263321

H,0,-2.7639169728,-1.7632568763,-2.6079758622

H,0,-1.1783890394,0.3497630861,-2.1176759532

H,0,-3.8332905773,1.9599419929,-0.6891344297

TS_1

(-1286.110408)

Fe,0,-2.6280726091,-0.456116754,-0.4196605554

C,0,-0.5809367958,-0.6057305831,-0.2943657225

C,0,-1.1803287815,-1.8810704301,-0.0175557074

C,0,-1.9156047618,-2.2716916154,-1.1695664534

C,0,-1.7789605998,-1.2477064068,-2.1574669675

C,0,-0.9530906614,-0.2184962161,-1.6247935313

C,0,-3.6783363719,1.3360485335,-0.360582924

C,0,-4.5081542894,0.2991436114,-0.8860911081

C,0,-4.6209096356,-0.7203267666,0.1065109338

C,0,-3.8608610935,-0.3148496511,1.2456346715

C,0,-3.2787341509,0.9556515958,0.9563851952

H,0,-1.0860662878,-2.4242170775,0.9124638677

H,0,-2.5063201062,-3.1729903079,-1.2674451468

H,0,-4.9503038628,0.2763469234,-1.8737995158

H,0,-5.1621382951,-1.6517712952,0.0015765303

H,0,-3.7205933749,-0.8842389262,2.1550344735

H,0,-2.6124639271,1.5075510512,1.6064250903

C,0,0.2394938343,0.1067300487,0.6855556355

C,0,3.2395388628,-0.2942333933,0.8502830314

C,0,2.2872723934,-1.3811245057,-0.9951542023

C,0,3.3529243559,-2.2072996146,-1.2056383029

H,0,1.4223072249,-1.4460528795,-1.6507919221

C,0,4.4928667751,-2.1334003371,-0.3195476518

H,0,3.370705429,-2.9248915629,-2.0161026111

N,0,2.1851783937,-0.4371963677,-0.0168561633

N,0,4.3193890267,-1.1491530911,0.6757648616

O,0,3.291166885,0.5628519939,1.7552193105

O,0,0.2765665742,0.025649452,1.8723974524

H,0,5.0868108145,-1.0278038545,1.3266262902

O,0,5.5222082571,-2.8003836315,-0.3576604692

O,0,0.3555342411,1.6596498891,0.0975146024

C,0,1.3197554802,2.2851143785,0.6538912867

H,0,1.9776102742,1.7481979596,1.3582424769

N,0,1.5971173321,3.5428526288,0.3682714892

C,0,2.7556893559,4.1837484474,0.9951428793

H,0,3.4217581155,4.5741270385,0.2185193208

H,0,2.4300259212,5.012220047,1.6335270351

H,0,3.2967419572,3.4465103796,1.5908565296

C,0,0.8176139096,4.3323818562,-0.582724705

H,0,-0.0211408392,3.7378015506,-0.9419345576

H,0,0.4446546119,5.2369372925,-0.0907194836

H,0,1.4499324473,4.6242114536,-1.4282479453

H,0,-2.2491189374,-1.2397094315,-3.1321821641

H,0,-0.6669001569,0.6994998395,-2.1165348735

H,0,-3.3824639345,2.2378367287,-0.8807232799

TS$_3$

(-1286.098915)

Fe,0,-2.7529102554,-0.4330015018,0.0668976587

C,0,-0.7106228711,-0.7136434332,-0.0037778718

C,0,-1.3387479241,-1.8166065182,0.6707083742

C,0,-2.23253152,-2.4278545889,-0.2495943629

C,0,-2.1634149619,-1.7069642625,-1.482794044

C,0,-1.2240501449,-0.6542652906,-1.3358995505

C,0,-3.6951556213,1.3919958295,-0.2450155902

C,0,-4.635116071,0.3251610407,-0.3840360329

C,0,-4.6777711456,-0.3888805223,0.851159936

C,0,-3.764227908,0.2350525227,1.7548235978

C,0,-3.1575805448,1.3347631192,1.0768136086

H,0,-1.1621721848,-2.1016929099,1.6986205035

H,0,-2.8789975422,-3.2703017686,-0.0406313019

H,0,-5.1938766499,0.0815786175,-1.278542552

H,0,-5.274659897,-1.2684218742,1.0557229769

H,0,-3.5437781964,-0.087420838,2.7640055361

H,0,-2.388196735,1.9792676707,1.4814260511

C,0,0.230833468,0.1717294256,0.6780055095

C,0,3.1091165715,-0.3725073834,0.8738402572

C,0,2.1731973125,-1.5093968302,-1.0345650319

C,0,3.1986958862,-2.5526890862,-0.8829360994

C,0,4.1032602343,-2.4601506341,0.1123531223

H,0,3.2091658257,-3.3662088031,-1.5971063158

N,0,2.1198632392,-0.5193624181,-0.0492193885

N,0,4.0634046889,-1.396010424,0.9791201781

O,0,3.2392402091,0.6133236889,1.6227840881

O,0,0.4045026221,0.3488090937,1.8432297306

H,0,4.7817938808,-1.2447778363,1.6728887016

O,0,0.2829872928,1.5665639924,-0.2289521209

C,0,1.3404258566,2.2225216911,0.0461350361

H,0,2.0349008962,1.8369859609,0.8086946101

N,0,1.6452637048,3.3503927975,-0.5700673538

C,0,2.8814342475,4.0527274124,-0.2266610995

H,0,3.5026260224,4.1657033638,-1.1217387762

H,0,2.6528610165,5.04662787,0.173883136

H,0,3.4304011551,3.4750538347,0.5191844687

C,0,0.8073481776,3.9260077997,-1.6197880449

H,0,-0.04814663,3.2746708461,-1.7921161275

H,0,0.460452356,4.9196305569,-1.3154783576

H,0,1.3885766168,4.0198366466,-2.5432632343

H,0,-2.743968683,-1.9147225271,-2.3720717072

H,0,-0.9286098235,0.0581392708,-2.089046595

H,0,-3.4190409384,2.0999770227,-1.0155691052

O,0,1.3960134299,-1.5090421899,-1.9928530005

H,0,4.8939909372,-3.184509553,0.2824626721

4a

(-1137.2219868)

Fe 2.061718 0.021359 -0.181434

C 0.405051 0.985922 0.603113

C 1.599685 1.388560 1.300330

C 2.468351 1.999594 0.359942

C 1.836592 1.963432 -0.922781

C 0.572610 1.334831 -0.782177

C 1.973032 -1.692577 -1.358774

C 3.235368 -1.045655 -1.525271

C 3.874825 -0.989357 -0.250643

C 3.009184 -1.601127 0.706705

C 1.836373 -2.035874 0.020492

H 1.779907 1.236205 2.355025

H 3.453866 2.393883 0.570320

H 2.261649 2.324479 -1.850235

H -0.138242 1.162202 -1.575810

H 1.240579 -1.869336 -2.135645

H 3.625358 -0.640477 -2.450131

H 4.834704 -0.534971 -0.041777

H 3.193155 -1.691801 1.769095

H 0.978421 -2.510791 0.478964

C -0.648049 0.216833 1.272665

C -2.569386 1.031994 -0.135460

C -2.566386 -1.198449 0.849509

C -3.760384 -1.525538 0.320039

H -2.018620 -1.847807 1.520141

C -4.462415 -0.586608 -0.545409

H -4.227673 -2.479199 0.526779

N -1.912839 0.002068 0.582254

N -3.764942 0.630440 -0.705246

O -2.142110 2.164987 -0.241125

H -4.248389 1.355406 -1.224631

O -5.537955 -0.759860 -1.091539

O -0.497640 -0.324701 2.352970

4a'

(-1137.21485174)

Fe -1.888169 -0.127500 -0.178390

C -0.143645 -1.109855 0.336938

C -1.280899 -1.777615 0.913021

C -2.116725 -2.206857 -0.150332

C -1.520926 -1.797503 -1.385417

C -0.308794 -1.119093 -1.092436

C -1.845868 1.799978 0.606478

C -1.919643 1.849201 -0.818360

C -3.115742 1.181706 -1.223112

C -3.778996 0.716166 -0.047585

C -2.993149 1.100020 1.083057

H -1.446099 -1.916884 1.972177

H -3.061737 -2.723753 -0.045099

H -1.939187 -1.949629 -2.371912

H 0.367442 -0.676548 -1.810862

H -1.020097 2.167477 1.199886

H -1.179020 2.291131 -1.471462

H -3.446942 1.026125 -2.241991

H -4.698689 0.145963 -0.020595

H -3.210164 0.867625 2.117554

C 0.924780 -0.501163 1.120866

O 0.954483 -0.393291 2.321735

C 2.248650 1.408539 0.191938

C 2.994486 -0.972248 -0.070987

C 3.492339 1.808193 -0.456825

C 4.375235 0.875605 -0.870870

H 3.670488 2.866745 -0.588833

H 5.312725 1.122184 -1.357629

N 2.084135 0.002778 0.327912

N 4.131377 -0.464889 -0.698838

H 4.802695 -1.165566 -0.982371

O 2.830531 -2.167108 0.100324

O 1.385706 2.180957 0.580132

References

1. Habjanec, L.; Frkanec, R.; Halassy, B.; Tomašić, J. Effect of liposomal formulations and immunostimulating peptidoglycan monomer (PGM) on the immune reaction to ovalbumin in mice. *J. Liposome Res.* **2006**, *16*, 1-16. https://doi/10.1080/08982100500528537
2. Halassy, B.; Krstanović, M.; Frkanec, R.; Tomašić, J. Adjuvant activity of peptidoglycan monomer and its metabolic products. *Vaccine* **2003**, *21*, 971. https://doi.org/10.1016/s0264-410x(02)00547-9
